# Shootins mediate collective cell migration and organogenesis of the zebrafish posterior lateral line system

**DOI:** 10.1038/s41598-019-48585-4

**Published:** 2019-08-21

**Authors:** Akihiro Urasaki, Seiya Morishita, Kosuke Naka, Minato Uozumi, Kouki Abe, Liguo Huang, Emiko Watase, Osamu Nakagawa, Koichi Kawakami, Takaaki Matsui, Yasumasa Bessho, Naoyuki Inagaki

**Affiliations:** 1 Laboratory of Systems Neurobiology and Medicine, Division of Biological Science, Nara Institute of Science and Technology, Ikoma, Nara 630-0192 Japan; 2 Department of Molecular Physiology, National Cerebral and Cardiovascular Center Research Institute, 6-1 Kishibe-Shinmachi, Suita, Osaka 564-8565 Japan; 3 Division of Molecular and Developmental Biology, National Institute of Genetics, and Department of Genetics, SOKENDAI (The Graduate University for Advanced Studies), Mishima, Shizuoka 411-8540 Japan; 4 Laboratory of Gene Regulation Research, Division of Biological Science, Nara Institute of Science and Technology, Ikoma, Nara 630-0192 Japan

**Keywords:** Development, Zebrafish

## Abstract

The zebrafish sensory posterior lateral line is an excellent model system to study collective cell migration and organogenesis. Shootin1 is a cytoplasmic protein involved in neuronal polarization and axon guidance. Previous studies have shown that shootin1 couples actin filament retrograde flow with extracellular adhesive substrates at the leading edge of axonal growth cones, thereby producing mechanical force for the migration and guidance of axonal growth cones. However, the functions of shootin in peripheral cells remain unknown. Here we identified two novel shootin family members, shootin2 and shootin3. In zebrafish, *shootin1* and *shootin3* are expressed in the posterior lateral line primordium (PLLP) and neuromasts during embryonic development. A *shootin1* mutant displayed a reduced speed of PLLP migration, while *shootin1*;*shootin3* double mutation inhibited cell proliferation in the PLLP. Furthermore, our results suggest that shootin1 and shootin3 positively regulate the number of neuromasts and the number of cells in deposited neuromasts. Our study demonstrates that shootins mediate collective cell migration of the posterior lateral line primordium and formation of neuromasts in zebrafish.

## Introduction

The lateral line of fish and amphibians is a mechanosensory system that senses directional water movements^1–3^. It is composed of two major branches, an anterior part that distributes on the head and a posterior part that extends on the trunk and tail. The individual sensory organs that constitute the lateral line, called neuromasts, are rosette shaped organs that contain the sensory hair cells and surrounding supporting cells. In zebrafish, the posterior lateral line is originated from the posterior lateral line primordium (PLLP). During lateral line formation, PLLP is formed just posterior to the otic vesicle and migrates toward the tail and periodically deposits cell clusters along the embryonic trunk; the cell clusters subsequently differentiate into neuromasts^1–3^. As these cell behaviors can be observed directly in the living embryo, the zebrafish PLL is an excellent model system for studying collective cell migration and organogenesis^1–6^.

Collective cell migration of the PLLP is regulated by multiple signaling pathways, including chemokine, Wnt and Fgf ^2,3,7–9^. Chemokine Cxcl12a and its receptors Cxcr4b and Cxcr7b are key regulators of PLLP migration^10–15^. The migrating PLLP consists of leading and trailing regions^16^; the leading cells express Cxcr4b and can respond to Cxcl12a, whereas the trailing cells, which express Cxcr7b, cannot respond to it^12,13^. Wnt signaling activated in the leading region restricts Cxcr7b expression and Fgf signaling to the trailing region^17^. Self-generated Cxcl12a gradients along the PLLP are required for proper migration of the PLLP^14,15^. In addition, notch signaling regulates the organogenesis of the PLL system: it acts downstream of Fgf signaling and increases the number of cells in the deposited neuromasts^18^. On the other hand, actin and actin-related proteins play key roles in cell morphogenesis and migration as readouts of various signaling pathways^19–22^. However, the molecular mechanics underlying PLLP migration and neuromast formation remain unclear.

Actin filaments (F-actins) polymerize at the leading edge of the motile cells and depolymerize proximally, which, in conjunction with myosin II activity, induces retrograde flow of F-actins^23–25^. Shootin1^26^, recently renamed shootin1a^27^, is a “clutch” molecule involved in neuronal polarization and axon guidance^26,28–30^. In cultured rat hippocampal neurons, shootin1a accumulates at the leading edge of axonal growth cones (Supplementary Fig. S1)^26^. It couples mechanically the F-actin retrograde flow (red arrow, Supplementary Fig. S1) and extracellular adhesive substrates, a process called clutch coupling, thereby transmitting the force of F-actin flow (red arrow) to the substrates as a traction force (white arrow)^31,32^. The driving force for growth cone advance (blue arrow) is produced as a counterforce to the traction forces exerted on the substrate (white arrow). In addition, the growth cone chemoattractant netrin-1 positively regulates shootin1a-mediated clutch coupling, through Pak1-mediated shootin1a phosphorylation, for axon outgrowth and guidance^33,34^. This coupling also contributes to the intracellular transport of actin and actin-binding proteins toward the leading edge of axons^35,36^. In addition to neuron-specific shootin1a, a splicing isoform shootin1b is produced from the *shootin1* gene, which is expressed both in the brain and in peripheral tissues^27^. A recent study reported that shootin1b-mediated clutch coupling produces force for migration of mouse olfactory interneurons^37^. In zebrafish, a homolog of mammalian *shootin1* has been reported in the genome^26^ whose expression is upregulated in the brain and peripheral tissues by knockdown of semaphorin or plexin^38^. However, the functions of shootin in peripheral cells are unknown.

In the present study, we identified two novel shootin family members, shootin2 and shootin3. We demonstrate that the three shootins are present in zebrafish; all of them interact with F-actin retrograde flow, suggesting that these proteins may function as clutch molecules. In addition, we show that *shootin1* and *shootin3* are expressed in the PLLP and neuromasts of zebrafish embryos. Loss of *shootin1* and *shootin3* reduced the speed of primordium migration, the number of neuromasts and the number of cells in deposited neuromasts. The *shootin1*;*shootin3* double mutation also inhibited cell proliferation in the PLLP. Overall, our findings indicate that shootins are involved in collective cell migration and formation of neuromasts during development of the zebrafish posterior lateral line system.

## Results

### Identification of novel shootin genes *shootin2* and *shootin3*

**Figure 1:**
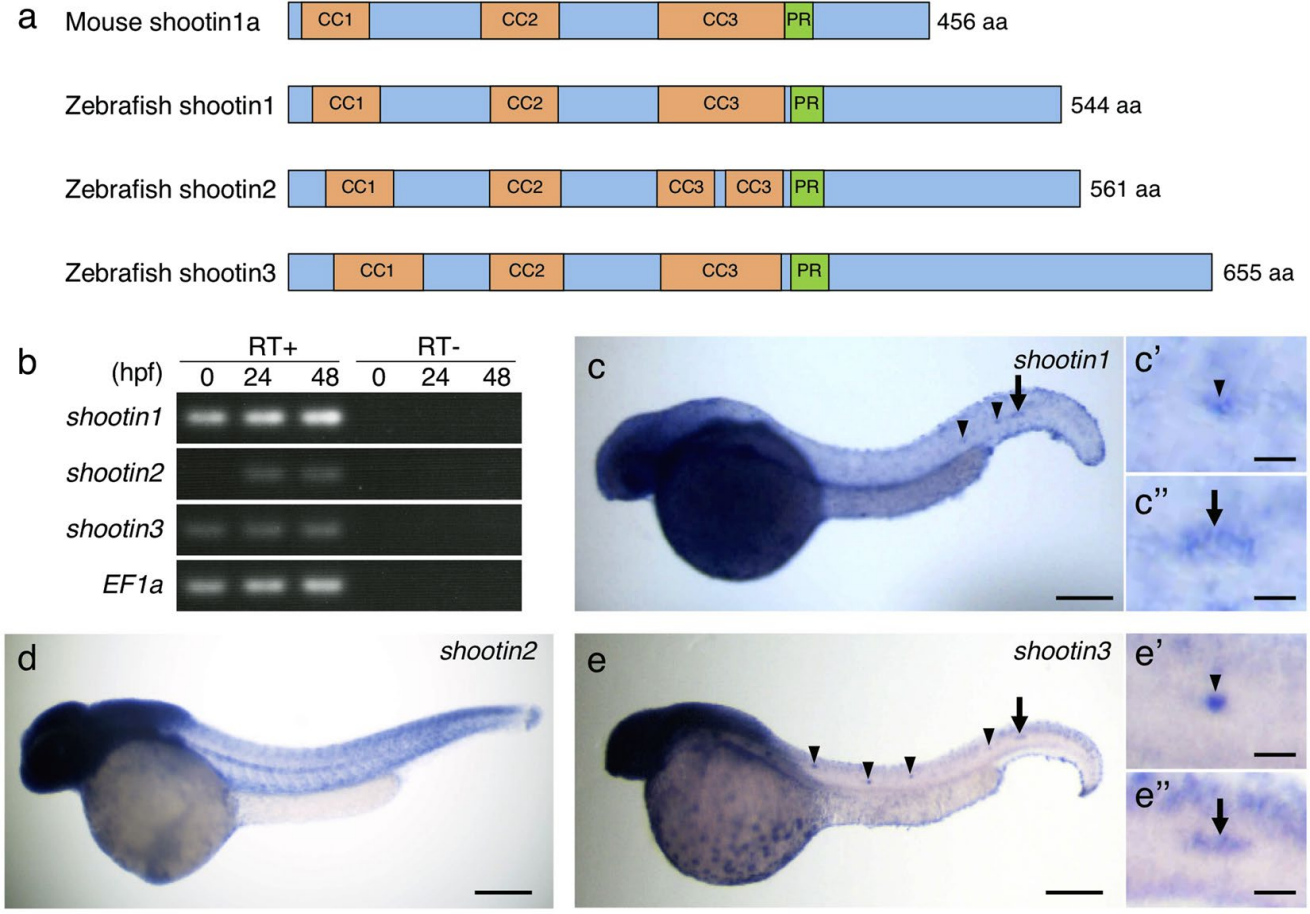
Identification and expression of zebrafish shootin genes. (**a**) Schematic representation of zebrafish shootin1, shootin2, shootin3 and mouse shootin1 protein structures. The top box for mouse shootin1 indicates the structural domains described previously^26^, and the bottom three boxes for zebrafish shootin1, shootin2 and shootin3 indicate the corresponding structural domains. CC1-3, coiled-coil domains 1–3; PR: proline-rich domain. (**b**) RT-PCR analysis of *shootin1*, *shootin2* and *shootin3* transcripts. Elongation factor 1a (EF1a) was used as a control. Developmental stages are denoted in hours post-fertilization (hpf). RT-PCR products produced in the presence (RT+) or absence (RT−) of reverse transcriptase were electrophoresed using a 3% agarose gel. (**c**–**e**) Whole-mount *in situ* hybridization of *shootin1* (**c**), *shootin2* (**d**) and *shootin3* (**e**) in zebrafish embryos at 36 hpf. Arrowheads and arrows indicate neuromasts and PLLP, respectively. Enlarged images indicate expression of *shootin1* and *shootin3* in the last deposited neuromasts (c’ and e’) and the PLLP (c” and e”). Scale bars: 200 μm for whole body images and 25 μm for enlarged images.

Shootin1a was originally identified in cultured rat hippocampal neurons^26^. Homologs of the rat *shootin1* gene have been identified in human, mouse and zebrafish genomes^26,38^. Here, we identified, by homology search analysis, two predicted shootin1-like proteins in zebrafish (XP_690881.3 and XP_685212.4). To analyze shootin1 and shootin1-like proteins in zebrafish embryos, we cloned their cDNAs. These cDNAs encode 544-, 561-, and 655-amino acid (aa) proteins that share high similarity with mouse shootin1a (Fig. 1a and Supplementary Fig. S2). Phylogenetic and synteny analyses revealed that shootins are clustered into three separate groups and that the gene encoding the 544-aa protein is orthologous to human *shootin1* (Supplementary Fig. S3a,b). We designated the genes encoding 561- and 655-aa proteins as *shootin2* and *shootin3*, respectively (Fig. 1a and Supplementary Fig. S2). The zebrafish *shootin1*, *shootin2* and *shootin3* genes are located on chromosome 17, 7 and 14, respectively (Supplementary Fig. S3b). Rat shootin1a is phosphorylated at Ser101 and Ser249 by Pak1 and these phosphorylations promote axon outgrowth^32,33^. The putative phosphorylation site corresponding to Ser101 is present in all the three zebrafish shootin proteins, whereas that corresponding to Ser249 is present in shootin1 and shootin3 but not in shootin2 (arrowheads, Supplementary Fig. S2).

Shootin1-homologous sequences were identified widely in vertebrates, including fish, frogs, reptiles, birds, marsupials and mammals (Supplementary Fig. S3a). Shootin2-homologous sequences were identified in fish, frog and Tasmanian devil (marsupial) but not in placental (eutherian) mammals (Supplementary Fig. S3a). In contrast, shootin3-homologous sequences were identified in fish, such as cavefish, medaka, fugu and coelacanth, but not in other vertebrate lineages (Supplementary Fig. S3a). Because a whole-genome duplication event occurred in the teleost lineage^39–43^, two orthologs of a gene are often identified in the zebrafish genome, whereas a single copy of the corresponding gene is present in the human genome. Synteny analysis showed that the locus containing zebrafish *shootin1* is conserved widely in vertebrates, such as human, mouse, Tasmanian devil, *Xenopus* and zebrafish (Supplementary Fig. S3b), whereas the locus containing zebrafish *shootin2* is conserved in Tasmanian devil, *Xenopus* and zebrafish (Supplementary Fig. S3b). The orders of genes in the loci containing *shootin2* and *shootin3* were different from those in the loci containing *shootin1*, suggesting that shootin2 and shootin3 are novel shootin family members.

### *shootin1* and *shootin3* are expressed in the zebrafish lateral line system

To examine whether the shootin family members are expressed in developing zebrafish embryos, we performed RT-PCR using specific primers. All three shootin genes were expressed at 24 h post-fertilization (hpf) and 48 hpf (Fig. 1b). The transcripts of *shootin1* and *shootin3* were detected at 0 hpf, while we could not detect *shootin2* at 0 hpf. Whole-mount *in situ* hybridization detected all the three shootin genes expressed in the anterior region of embryos at 36 hpf (Fig. 1c–e). In addition, *shootin1* and *shootin3* were expressed in the neuromasts (arrowheads, Fig. 1c,c’,e,e’) and the PLLP (arrows, Fig. 1c,c”, e, e”) of the lateral line system.

### Zebrafish shootins interact with F-actin retrograde flow at the cellular leading edge

**Figure 2:**
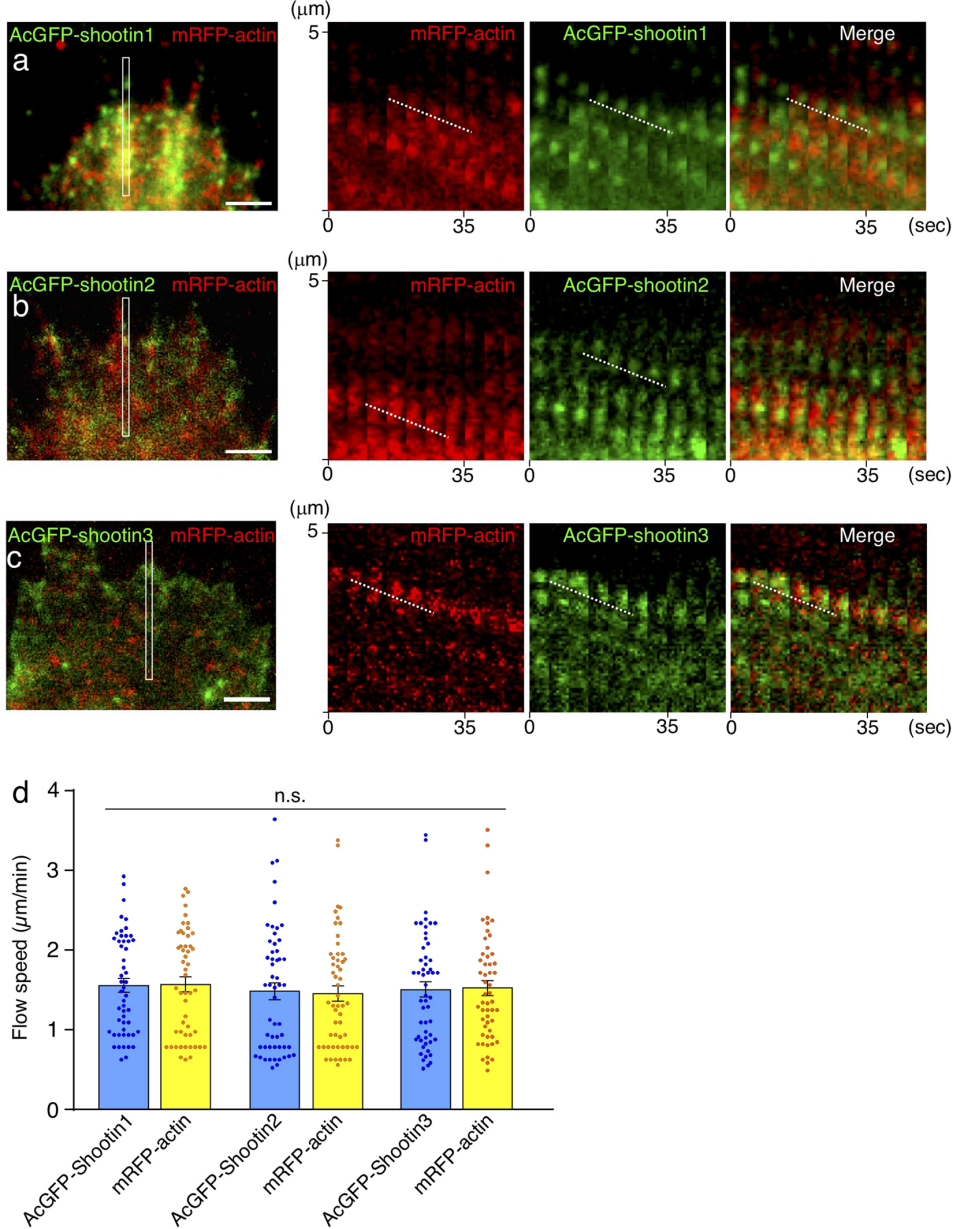
Zebrafish shootin1, shootin2 and shootin3 interact with F-actin retrograde flow. (**a**–**c**) Fluorescent speckle images of AcGFP-shootin1 (**a**), AcGFP-shootin2 (**b**) and AcGFP-shootin3 (**c**) with mRFP-actin in XTC fibroblasts (see Movies 1–3). Kymographs (right) of the areas indicated by rectangles in the left panels show that the fluorescent features of AcGFP-shootin and those of mRFP-actin moved at similar speed (dotted lines). (**d**) Retrograde flow speeds of shootin1 (*n* = 51 speckles), shootin2 (*n* = 54 speckles), shootin3 (*n* = 53 speckles) and F-actin (*n* = 107 speckles) measured from the kymograph analysis in (**a**–**c**). Scale bars: 2 μm.

Previous speckle imaging analyses *in vitro* showed that shootin1a interacts with F-actin retrograde flow at the leading edge of axonal growth cones, and demonstrated that the shootin1a–F-actin interaction promotes growth cone migration^31,32^ (Supplementary Fig. S1). We next examined whether the zebrafish shootins interact with F-actin retrograde flow. The speckle imaging assay is a useful method to monitor the retrograde flows of F-actin and clutch molecules^32,44^. As speckle imaging analysis of F-actin flow *in vivo* is technically difficult, we examined shootin dynamics using cultured XTC fibroblasts, a cell line established from *Xenopus laevis*^45^. XTC fibroblasts bear large and thin lamellipodia, and are thus particularly suitable for speckle imaging of the retrograde flows of F-actin and clutch molecules^32,44^. AcGFP-shootin1 and mRFP-actin were coexpressed in XTC fibroblasts; speckle imaging was performed as described^31^. The AcGFP-shootin1 signals moved retrogradely in the lamellipodia of XTC fibroblasts (Fig. 2a and Movie 1), as reported in the case of mammalian shootin1a^31^. Speckles of AcGFP-shootin1 moved with those of mRFP-actin at a similar speed (AcGFP-shootin1: 1.56 ± 0.09 µm/min, *n* = 51 speckles; mRFP-actin: 1.57 ± 0.09 µm/min, *n* = 51 speckles; Fig. 2a,d, Movie 1). Speckles of AcGFP-shootin2 and AcGFP-shootin3 also underwent retrograde movement at the leading edge of XTC fibroblasts. The speed of AcGFP-shootin2 speckles was similar to that of mRFP-actin speckles (AcGFP-shootin2: 1.48 ± 0.11 µm/min, *n* = 54 speckles; mRFP-actin: 1.46 ± 0.11 µm/min, *n* = 54 speckles; Fig. 2b,d, Movie 2); the AcGFP-shootin3 speckles also moved with mRFP-actin at a similar speed (AcGFP-shootin3: 1.51 ± 0.11 µm/min, *n* = 53 speckles; mRFP-actin: 1.52 ± 0.09 µm/min, *n* = 53 speckles; Fig. 2c,d, Movie 3). These results indicate that zebrafish shootin1, shootin2 and shootin3 interact with F-actin retrograde flow at the cellular leading edge.

### Zebrafish shootin1 mediates PLLP migration

**Figure 3:**
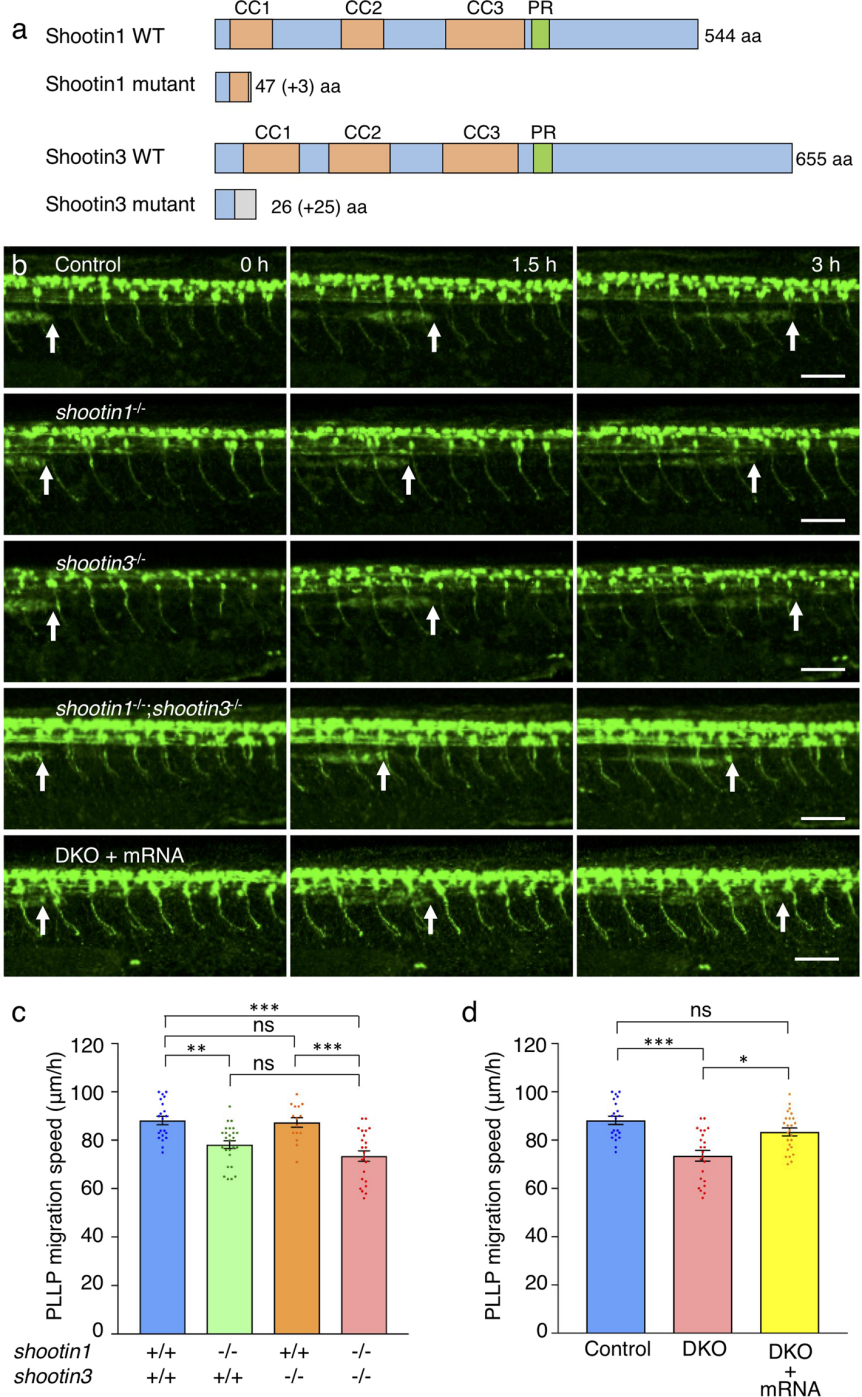
*shootin1* mutants display reduced migration speed of the PLLP. (**a**) Schematic structures of shootin1 and shootin3 proteins of the *shootin1* and *shootin3* single mutants. Frameshift mutations in *shootin1* and *shootin3* resulted in premature stop codons after amino acid positions 47 and 26, respectively. The gray boxes indicate amino acids added by the frameshift mutations; numbers in brackets indicate the numbers of these additional residues. CC1-3: coiled-coil domain; PR: proline-rich domain. (**b**) Representative time-lapse images of wild-type control, *shootin1*^−/−^ single mutant, *shootin3*^−/−^ single mutant and *shootin1*^−/−^;*shootin3*^−/−^ double mutant embryos carrying the SAIGFF213A;UAS:GFP construct. Time-lapse images of a *shootin1*^−/−^;*shootin3*^−/−^ double mutant embryo into which *shootin1* and *shootin3* mRNAs were injected are also presented at the bottom. Arrows indicate the leading edges of PLLPs. Scale bars: 50 μm. (**c**) Migration speeds of PLLP in wild-type control (*n* = 21), *shootin1*^−/−^ single mutant (*n* = 26), *shootin3*^−/−^ single mutant (*n* = 15) and *shootin1*^−/−^;*shootin3*^−/−^ double mutant (*n* = 23) embryos at 32–38 hpf obtained from the analyses in (**b**). (**d**) Rescue analyses of the PLLP migration in the *shootin1*;*shootin3* double mutants. *shootin1* and *shootin3* mRNAs were injected into the *shootin1*^−/−^;*shootin3*^−/−^ double knockout (DKO) mutant embryos (DKO + mRNA, *n* = 25). Data for the uninjected wild-type (WT) and DKO mutant embryos in (**d**) are shared with those in (**c**). Data for (**c**) and (**d**) represent mean ± SEM. Statistical significance of the differences is indicated with asterisks (***P < 0.01; **P < 0.02; *P < 0.05; ns, nonsignificant).

The interactions of zebrafish shootin family members with F-actin retrograde flow in XTC fibroblasts raise the possibility that shootin1 and shootin3 mediate the migration of the posterior lateral line system. To assess the functions of shootin1 and shootin3 in the posterior lateral line, we generated *shootin1* and *shootin3* mutants using the CRISPR/Cas9 system. The *shootin1* mutant allele contained an 8-bp deletion in the third exon, resulting in a frame shift and a premature stop codon after 47 aa (Fig. 3a and Supplementary Fig. S4a,b). The *shootin3* mutant allele harbored a 13-bp deletion in the second exon, resulting in a premature stop codon after 26 aa (Fig. 3a and Supplementary Fig. S5a,b). We identified *shootin1* mutants by PCR-based genotyping (Supplementary Fig. S4c) and *shootin3* mutant fish by T7 endonuclease I (T7EI)-based genotyping (Supplementary Figs S5c and S6), respectively. To visualize the PLLP, we crossed the fish with SAIGFF213A;UAS:GFP transgenic fish that express GFP in the PLLP^46^. Consistent with a previous report^47^, during 32–38 hpf, PLLP migrated at 88 ± 2 μm/h in control embryos (*n* = 21) (Movie 4, Fig. 3b). PLLP also migrated in the *shootin1* mutant embryos (Movie 5, Fig. 3b); however, the migration speed was significantly slower than that in the control embryos (Fig. 3c). On the other hand, no significant difference in the migration speed of PLLP was detected between control and *shootin3* mutant embryos (Movies 4 and 6, Fig. 3b,c).

We also generated a *shootin1*;*shootin3* double mutant. As in the case of *shootin1* single mutant, the PLLP migration speed was significantly lower than that in control embryos and *shootin3* single mutant embryos (Movies 4, 6 and 7, Fig. 3b,c). When *shootin1* and *shootin3* mRNAs were injected into the double mutant embryos, the reduced speed of PLLP migration was rescued to a level similar to that in the control embryos (Fig. 3b,d). Injection of *shootin1* mRNA into the *shootin1* single mutant embryos also rescued the reduced speed of PLLP migration (Supplementary Fig. S7). The migrating PLLP consists of leading and trailing regions^16^; Cxcr4b is expressed in all the cells of the PLLP, whereas Cxcr7b is expressed exclusively in the trailing cells^1^. We further examined the polarity of PLLP in mutant embryos, using Cxcr4b and Cxcr7b as markers. As shown in Supplementary Fig. S8a, *cxcr4b* was expressed widely in the wild-type PLLP, whereas *cxcr7b* was expressed only in the trailing region. Their expression patterns in the *shootin1*;*shootin3* double mutant PLLP were similar to those in the wild-type PLLP, suggesting that PLLP polarity was not affected by the *shootin1*;*shootin3* double mutation. Taking these data together, we conclude that shootin1 plays a key role in PLLP migration.

### Shootin1 and shootin3 mediate neuromast formation

**Figure 4:**
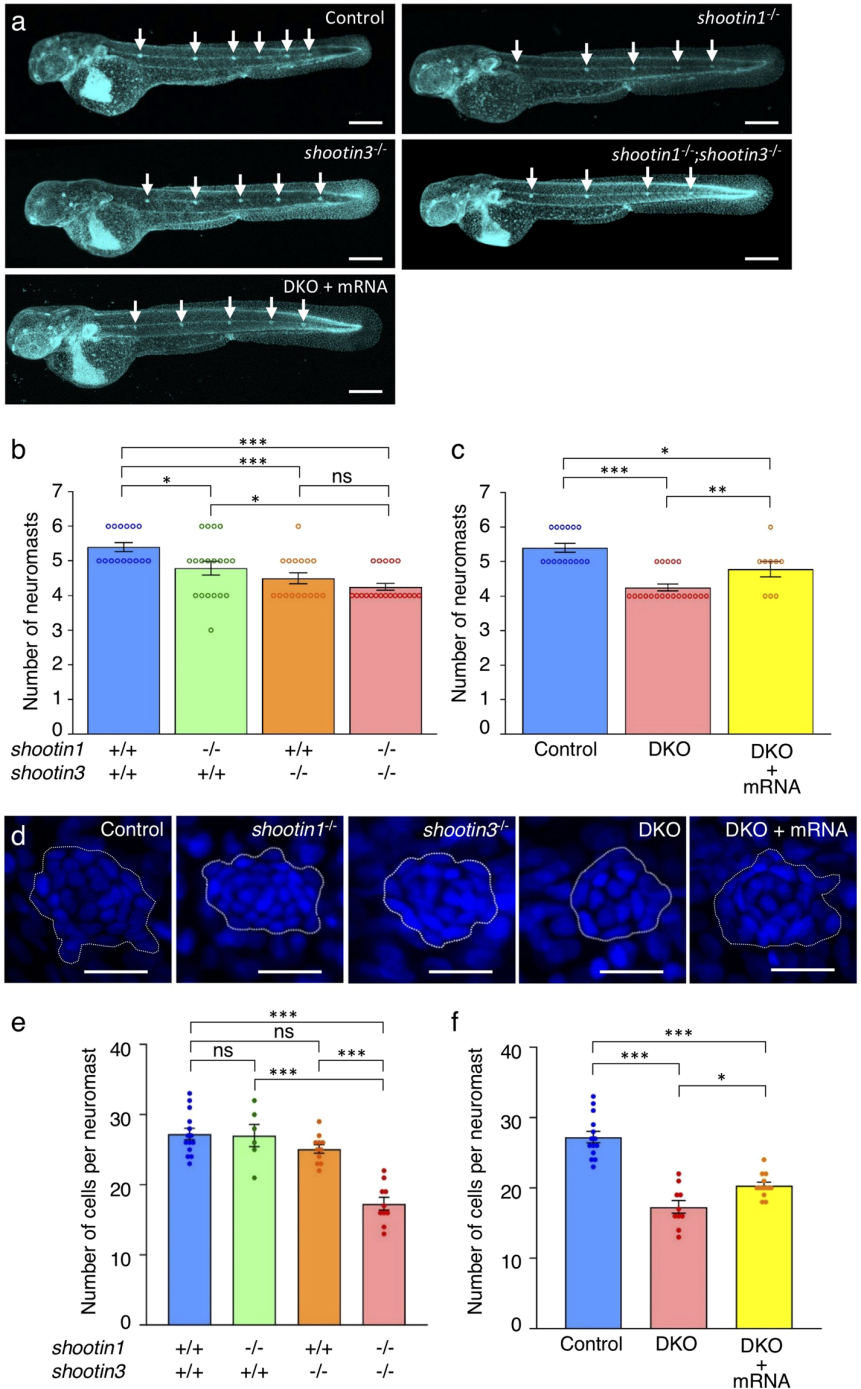
*shootin1*;*shootin3* double mutants exhibit reduced number of neuromasts and reduced number of cells in neuromasts. (**a**) Representative images of wild-type control, *shootin1*^−/−^ single mutant, *shootin3*^−/−^ single mutant and *shootin1*^−/−^;*shootin3*^−/−^ double mutant embryos at 48 hpf. An image of a *shootin1*^−/−^;*shootin3*^−/−^ double mutant embryo into which *shootin1* and *shootin3* mRNAs were injected is also presented at the bottom. Embryos were stained with DAPI. Scale bars: 300 μm. (**b**) The number of neuromasts obtained from the analyses in (**a**). The neuromast numbers of wild-type control (*n* = 15), *shootin1*^−/−^ single mutant (*n* = 19), *shootin3*^−/−^ single mutant (*n* = 16) and *shootin1*^−/−^;*shootin3*^−/−^ double mutant (*n* = 20) embryos were counted at 48 hpf. (**c**) Rescue experiments of the reduced number of neuromasts in the *shootin1*;*shootin3* double mutants. Injected DKO embryos (DKO + mRNA, *n* = 9) were observed at 48 hpf. Data for the uninjected wild-type (WT) and DKO mutant embryos in (**c**) are shared with those in (**b**). (**d**) Representative images of the DAPI-stained first deposited neuromasts in wild-type control, *shootin1*^−/−^ single mutant, *shootin3*^−/−^ single mutant and *shootin1*^−/−^;*shootin3*^−/−^ double mutant embryos at 32 hpf. An image of a neuromast in *shootin1*^−/−^;*shootin3*^−/−^ double mutant embryo into which *shootin1* and *shootin3* mRNAs were injected is also shown to the right. Dotted lines indicate the areas of neuromasts, in which small nuclei of neuromast cells cluster. Scale bars: 20 μm. (**e**) The number of cells in the first deposited neuromasts obtained from the analyses in (**d**). Wild-type control (*n* = 14), *shootin1*^−/−^ single mutant (*n* = 6), *shootin3*^−/−^ single mutant (*n* = 11), and *shootin1*^−/−^;*shootin3*^−/−^ double mutant (*n* = 10) embryos were analyzed at 32 hpf. (**f**) Rescue experiments of the reduced cell number of the double mutant neuromasts. Injected DKO embryos (DKO + mRNA, *n* = 12) were observed at 32 hpf. Data for the uninjected wild-type (WT) and DKO mutant embryos in (**f**) are shared with those in (**e**). Data in (**b**,**c**,**e** and **f**) represent mean ± SEM; ***P < 0.01; *P < 0.05; ns, nonsignificant.

Next, we analyzed the number of neuromasts in control and mutant fish after PLLP migration (48 hpf) (Fig. 4). Consistent with previous reports^17,47^, the average number of neuromasts was 5.4 ± 0.1 (*n* = 15 embryos) in control embryos, and it was significantly reduced by *shootin1* and *shootin3* single mutations (Fig. 4a,b). The number of neuromasts was further decreased in the *shootin1*;*shootin3* double mutants (Fig. 4a,b). Moreover, the injection of *shootin1* and *shootin3* mRNAs rescued significantly the reduced number of neuromasts in the double mutants (Fig. 4a,c). We further counted the number of cells in the first deposited neuromasts at 32 hpf by DAPI staining^18,48,49^ (Fig. 4d). In control embryos, the number of cells detected in the neuromasts was 27.2 ± 0.8 (*n* = 14 neuromasts), which is similar to the previously reported number^18^. The *shootin1*;*shootin3* double mutants exhibited a reduced number of neuromast cells, although no significant differences were observed between the control and single mutants (Fig. 4d,e). Furthermore, the injection of *shootin1* and *shootin3* mRNAs rescued partially the reduced cell number in the double mutants (Fig. 4d,f). These data indicate that shootin1 and shootin3 mediate neuromast formation, by positively regulating both the neuromast number and the number of cells in neuromasts.

### *shootin1*;*shootin3* double mutation reduces cell proliferation in the PLPP

**Figure 5:**
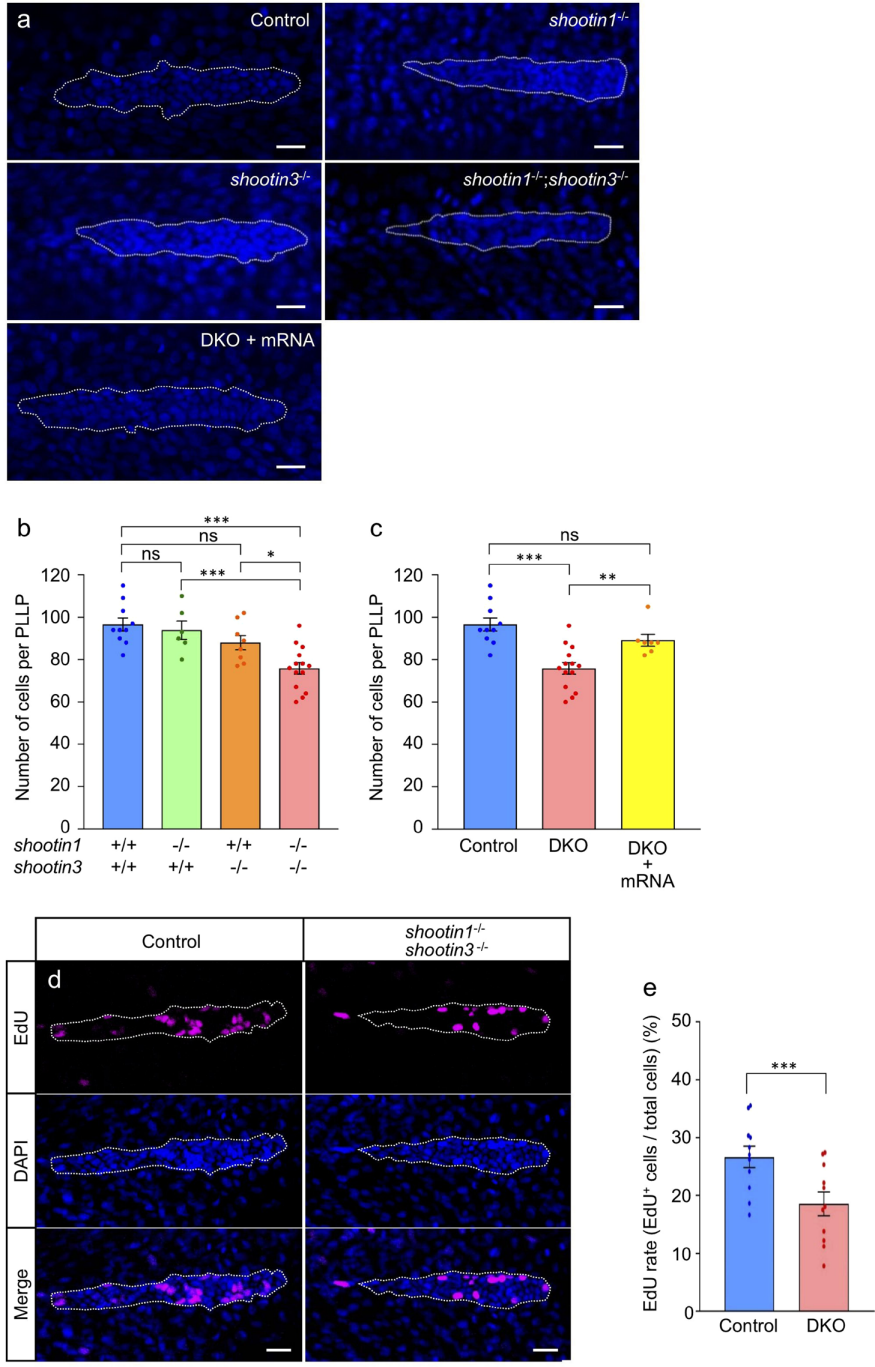
*shootin1*;*shootin3* double mutants exhibit reduced cell number and reduced cell proliferation in the PLLP. (**a**) Representative images of DAPI-stained PLLP in wild-type control, *shootin1*^−/−^ single mutant, *shootin3*^−/−^ single mutant and *shootin1*^−/−^;*shootin3*^−/−^ double mutant embryos at 32 hpf. An image of a PLLP in *shootin1*^−/−^;*shootin3*^−/−^ double mutant embryo into which *shootin1* and *shootin3* mRNAs were injected is also presented at the bottom. Dotted lines indicate the areas of PLLP, in which small nuclei of PLLP cells cluster. Scale bars: 20 μm. (**b**) Number of cells in the PLLP of wild-type control (*n* = 10), *shootin1*^−/−^ single mutant (*n* = 6), *shootin3*^−/−^ single mutant (*n* = 8) and *shootin1*^−/−^;*shootin3*^−/−^ double mutant (*n* = 14) embryos at 32 hpf obtained from the analyses in (**a**). (**c**) Rescue experiments of the reduced cell number of double mutant PLLP cells. The injected DKO embryos (DKO + mRNA, *n* = 7) were analyzed at 32 hpf. Data for the uninjected wild-type (WT) and DKO mutant embryos in (**c**) are shared with those in (**b**). (**d**) Representative images of PLLP stained with EdU and DAPI in wild-type control and *shootin1*^−/−^; *shootin3*^−/−^ mutant embryos at 32 hpf. Scale bars: 20 µm. (**e**) Ratio of EdU-positive cells relative to the total number of cells in the WT (*n* = 11) and DKO mutant (*n* = 11) PLLP at 32 hpf obtained from the analyses in (**d**). Data in (**b**), (**c**) and (**e**) represent mean ± SEM; ***P < 0.01; *P < 0.05; ns, nonsignificant.

To investigate how shootin1 and shootin3 mediate neuromast formation, we quantified the number of cells in the PLLP at 32 hpf using DAPI staining^18,48,49^ (Fig. 5a). In control embryos, the number of cells detected in the PLLP was 96.6 ± 3.1 (*n* = 10 PLLPs), which is consistent with previous data^18^. Although no significant differences were observed in the mean cell numbers in the PLLP between the control and shootin single mutant fish, the *shootin1*;*shootin3* double mutants displayed significantly reduced PLLP cell numbers compared with the control (Fig. 5a,b). We also found that the average number of cells in the double mutant PLLP increased following the injection of *shootin1* and *shootin3* mRNAs (Fig. 5a,c). We further analyzed the PLLP cell number before the first neuromast deposition (24 hpf). In control embryos, the number of cells detected in the PLLP was 125.6 ± 2.7 (*n* = 7 PLLPs). As shown in Supplementary Fig. S9, the cell numbers of the single and double mutant PLLP were similar to those of the wild-type PLLP at 24 hpf.

As previous studies have shown that cells in the migrating PLLP proliferate^47,50,51^, we next analyzed cell proliferation by EdU labelling (Fig. 5d). The ratio of EdU-positive proliferating cells to the total number of cells in the PLLP was 26.7 ± 1.9% (*n* = 11 PLLPs) (Fig. 5d,e). The ratio of EdU-positive proliferating cells was reduced significantly by the *shootin1*;*shootin3* double mutation (Fig. 5d,e). We finally analyzed cell fate determination of hair cells by examining the expressions of their markers *atoh1a* and *deltaA*^1,3^. Expressions of *atoh1a* and *deltaA* in the PLLP and first deposited neuromast were detected in wild-type and *shootin1*;*shootin3* double mutant embryos (Supplementary Fig. S8b). Their expression patterns in the double mutant were similar to those in the wild-type embryo, suggesting that hair cell fate determination was not affected by the *shootin1*;*shootin3* double mutation. Together, these data suggest that shootin1 and shootin3 positively regulate cell proliferation in the migrating PLLP.

## Discussion

We report here two novel shootin family members, shootin2 and shootin3. Phylogenetic analyses identified shootin1 in a wide range of vertebrates, including eutherians, marsupials, birds, reptiles, amphibians and fish. Shootin2 was found in marsupial, frog and fish genomes, while shootin3 is restricted to the fish lineage. Zebrafish *shootin1* and *shootin3* were expressed in the PLLP and neuromasts. The mutation in *shootin1* reduced the speed of primordium migration, while *shootin1*;*shootin3* double mutation led to reduction in cell proliferation in the primordium, neuromast number and number of cells in the neuromasts. Together, these results demonstrate key roles of shootins in collective cell migration and neuromast formation of the zebrafish posterior lateral line system.

**Figure 6:**
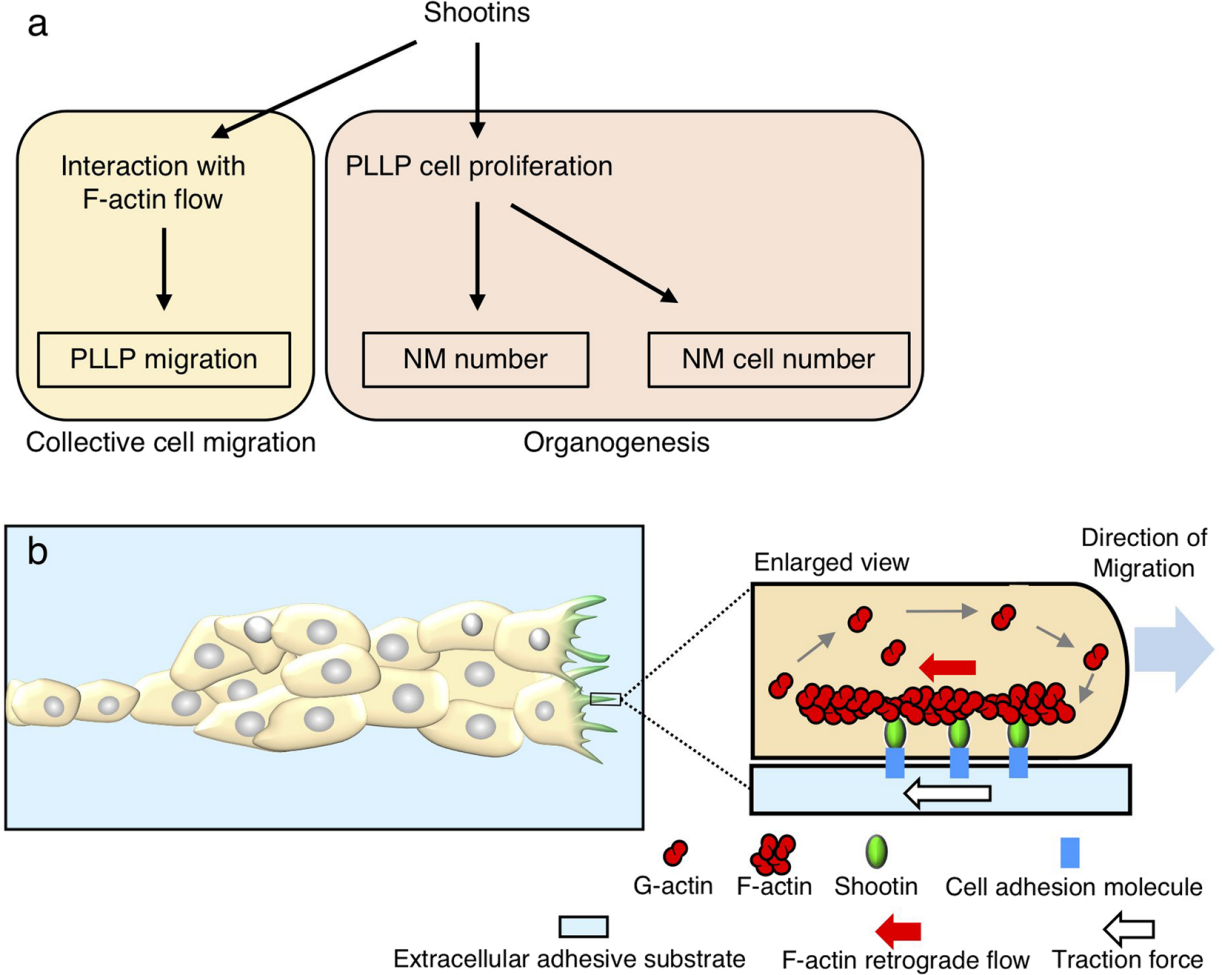
A working model for how shootins mediate collective cell migration and organogenesis of the zebrafish posterior lateral line system. (**a**) A model for shootin-mediated collective cell migration and organogenesis of the zebrafish posterior lateral line system. Shootins mediate PLLP migration through its interaction with F-actin at the leading edge of the leader cells. Shootins promote cell proliferation in the PLLP, thereby positively regulating the number of neuromasts and the number of cells in the deposited neuromasts. (**b**) A mechanistic model for shootin1-mediated zebrafish PLLP migration. F-actins polymerize at the filopodia and lamellipodia of PLLP leading cells and depolymerize proximally, thereby inducing retrograde flow of F-actins (red arrow). Shootin1 couples mechanically the F-actin retrograde flow and extracellular adhesive substrates, thereby transmitting the force of F-actin flow (red arrow) to the substrates as a traction force (white arrow)^31,32,34^. The driving force for PLLP migration (blue arrow) is produced as a counterforce to the traction forces exerted on the extracellular substrates (white arrow).

Concerning the mechanism for shootin1-mediated PLLP migration, our data indicate that zebrafish shootin1 interacts with F-actin retrograde flow at the leading edge of fibroblasts. Previous studies reported that rat shootin1a interacts with F-actin retrograde flow at the leading edge of axonal growth cones, thereby producing force for growth cone migration as a clutch molecule (Supplementary Fig. S1)^31–33^. A recent study also demonstrated that mouse shootin1b produces force for neuronal migration through its interaction with F-actin retrograde flow^37^. In the zebrafish PLLP, F-actin accumulates at the leading edge of the leader cells during collective cell migration^52^. Thus, we consider that zebrafish shootin1 is likely to mediate PLLP migration by interacting with F-actin at the leading edge of the leader cells (Fig. 6a,b). Although shootin3 interacted with F-actin retrograde flow, the *shootin3* mutation did not have a significant effect on PLLP migration. However, we do not rule out the possibility that shootin3 functions as a clutch molecule. As a possible reason to explain the difference between the phenotypes of *shootin1*^−/−^ and *shootin3*^−/−^ fish, the efficiency of shootin3-mediated clutch coupling may be weaker than that of shootin1-mediated clutch coupling. Alternatively, the expression level of shootin3 in PLLP may be lower than that of shootin1.

PLLP migration is regulated by the extracellular signal Cxcl12a and its receptors Cxcr4b and Cxcr7b^10–15^. In previous reports, we demonstrated that the extracellular chemoattractant netrin-1 induces PAK1-mediated shootin1a phosphorylation at Ser101 and Ser249 in the axonal growth cones; this in turn enhances the coupling between F-actin retrograde flow and cell adhesion, thereby promoting growth cone migration^32–34^. Interestingly, phosphorylatable serine residues corresponding to these sites are present in zebrafish shootin1, raising a possibility that PLLP migration may be positively regulated by phosphorylation at these sites under chemoattractant signals. Elucidating the detailed molecular mechanisms involved in shootin1-mediated PLLP migration remains an important issue for future investigation.

The present study demonstrated that the number of neuromasts as well as the number of cells in neuromasts were reduced in the *shootin1*;*shootin3* double mutant embryos. As the effect of the *shootin1*;*shootin3* double mutation was larger than that of *shootin1* single mutation or *shootin3* single mutation, we think that shootin1 and shootin3 up-regulate cooperatively the neuromast number and the number of cells in neuromasts. Concerning these results, our data also showed that the *shootin1*;*shootin3* double mutation inhibits cell proliferation in the PLLP, suggesting that shootin1 and shootin3 promote cell proliferation there. Although the molecular mechanisms underlying the shootin-mediated cell proliferation are unclear, we expect that the increased cell proliferation in the PLLP positively regulates both the number of neuromasts and the number of the cells in neuromasts (Fig. 6a). The details of how neuromast formation is regulated by shootin1 and shootin3 remain for further analyses.

## Methods

### Zebrafish and husbandry

All relevant aspects of the experimental procedures were approved by the Institutional Animal Care and Use Committee of Nara Institute of Science and Technology (reference No. 1321) and carried out in accordance with relevant guidelines and regulations. Wild type and the SAIGFF213A line^46^ were used in this study. Zebrafish were maintained under standard husbandry conditions at 28.5 °C with a 14 h light and 10 h dark cycle.

### Cloning of zebrafish shootin cDNAs

Total RNA was purified from zebrafish embryos using TRIzol reagent (Invitrogen), according to the manufacturer’s instructions. The RNA sample was used to synthesize cDNA with MLV RT (H-) Point Mutant (Promega) using primer AP (Supplementary Table S1) for reverse transcription. Specific cDNAs were PCR-amplified using the following primers: shootin1-h-Ba-Ko and shootin1-t-Xb-No for shootin1, shootin2-h-Ba-Ko and shootin2-t-Xb-No for shootin2, and shootin3-h-Ba-Ko and shootin3-t-Sp for shootin3 (Supplementary Table S1). The shootin1 and shootin2 fragments were digested with BamHI and XbaI restriction endonucleases, whereas shootin3 fragments were digested with BamHI and SpeI. The cDNAs were then cloned into the BamHI-XbaI site of pCS2 and sequenced using an ABI PRISM 3130 (Applied Biosystems).

### Vector construction

pCS2-EGFP-shootin1 was constructed as follows. pCS2-MCS was first constructed by self-ligating the PCR product amplified from pCS2 using the primers pCS2-5′-out-Ba and pCS2-3′-out-Ba-Sp-Bg-Xb (Supplementary Table S1), and digesting with BamHI. pCS2-EGFP was constructed by replacing a short BamHI-SpeI fragment of pCS2-MCS with the EGFP gene fragment PCR-amplified from pT2AL200R150G^53^ using the primers EGFP-h-Bg-Ko and EGFP-t-Sp (Supplementary Table S1), and digesting with BamHI and SpeI. Finally, pCS2-EGFP-shootin1 was constructed by replacing a short BglII-XbaI fragment of pCS2-EGFP with the shootin1 gene fragment amplified from shootin1 cDNA using the primers shootin1-h-Ba and shootin1-t-Xb-No (Supplementary Table S1), and digesting with BamHI and XbaI. pCS2-EGFP-shootin2 and pCS2-EGFP-shootin3 were constructed in a similar manner to pCS2-EGFP-shootin1, using primers shootin2-h-Ba and shootin2-t-Xb-No for shootin2, and primers shootin3-h-Ba and shootin3-t-Sp for shootin3 (Supplementary Table S1). For constructing pAcGFP-shootin1, a short BamHI-SalI fragment of pAcGFP-C1 (Clontech) was replaced with the shootin1 gene fragment, which was amplified from *shootin1* cDNA using shootin1-h-Ba and shootin1-t-Sa primers (Supplementary Table S1), and digested with BamHI and SalI. pAcGFP-shootin2 and pAcGFP-shootin3 were constructed in a similar manner to pAcGFP-shootin1, using shootin2-h-Ba and shootin2-t-Sa primers for *shootin2*, and shootin3-h-Ba and shootin3-t-Sa primers for *shootin3* (Supplementary Table S1).

### Identification of shootin genes and phylogenetic analysis

Shootin family members were identified in the zebrafish genome using BLAST analysis. Multiple alignments and phylogenetic analysis were performed using Genetyx ver.13 (Genetyx). The coiled-coil domains of shootin proteins were predicted using SMART^54^. The phylogenetic tree was constructed using the neighbor-joining method^55^.

### RT-PCR

To analyze the expression of shootin family members during zebrafish development, RT-PCR was performed using shootin1-rt-f and shootin1-rt-r primers for shootin1, shootin2-rt-f and shootin2-rt-r primers for shootin2, and shootin3-rt-f and shootin3-rt-r primers for *shootin3*. We used EF1a-rt-f and EF1a-rt-r primers as a positive control. The primers used are listed in Supplementary Table S1.

### DNA electrophoresis

The DNA electrophoresis in Fig. 1c and Supplementary Figs S4c and S5c was performed using agarose gels. The images in Fig. 1b were cropped from full-length gel images (Supplementary Fig. S10). All the gel images are raw data without modification.

### Whole-mount *in situ* hybridization

Whole-mount *in situ* hybridization was performed as described previously^56^. The following plasmids were constructed for synthesizing *in situ* probes. For constructing pCRII-shootin1, *shootin1* was PCR-amplified using the primers shootin1-h-Ba-Ko and shootin1-r2 and cloned into pCRII-TOPO (Invitrogen). For constructing pGEMT-shootin2, *shootin2* was PCR-amplified with shootin2-h4 and shootin2-r4 and cloned into pGEM-T (Promega). Similarly, to construct pGEMT-shootin3, *shootin3* was PCR-amplified with shootin3-f and shootin3-t-Sp and cloned into pGEM-T (Promega). Plasmids pCRII-shootin1 and pGEMT-shootin3 were digested with SpeI, and antisense probes of *shootin1* and *shootin3* were synthesized using T7 RNA polymerase (Roche). The plasmid pGEMT-shootin2 was digested with SacII, and *shootin2* antisense probes were synthesized using SP6 RNA polymerase (Roche). For constructing pCRII-*atoh1a*, pCRII-*cxcr4b*, pCRII-*cxcr7b* and pCRII-*deltaA*, *atoh1a*, *cxcr4b*, *cxcr7b* and *deltaA* were PCR-amplified with specific primer pairs and cloned into pCRII (Invitrogen). The plasmids pCRII-*atoh1a*, pCRII-*cxcr4b*, pCRII-*cxcr7b* and pCRII-*deltaA* were digested with NotI, and antisense probes of *atoh1a*, *cxcr4b*, *cxcr7b* and *deltaA* were synthesized using SP6 RNA polymerase (Roche). All *in situ* probes were synthesized from cDNAs using the DIG RNA labeling kit (Roche), according to the manufacturer’s instructions. Images were acquired with a Leica MZFL III or a Keyence BZ-X700/BZ-X710 microscope and processed using Adobe Photoshop Elements 12 and Fiji^57^.

### Fluorescent speckle microscopy

XTC fibroblasts, a cell line established from *Xenopus laevis*^45^, were cultured as described previously^58^ and transfected with pAcGFP-shootin1 or pAcGFP-shootin3 and pmRFP-actin^35^ using the X-treamGENE 9 transfection reagent (Sigma). Fluorescent speckle imaging was performed as described previously^31,32,44^. Images were acquired at 28 °C using an Observer.Z1 fluorescence microscope (Zeiss) equipped with an ORCA-flash4.0digital CMOS camera (Hamamatsu) and ZEN imaging software (Zeiss). Images were processed using Fiji^57^ and Adobe Photoshop Elements 12 as described previously^31,32^.

### Generation of zebrafish *shootin1* and *shootin3* mutants

Zebrafish mutants of *shootin1* and *shootin3* were generated using the CRISPR/Cas9 system^59^. Vectors for customized guide RNAs (gRNAs) were constructed as described previously^59^. Plasmid pT7-shootin1 (ex3) was constructed by cloning the two annealed oligonucleotides shootin1-f-ex3 and shootin1-r-ex3. pT7-shootin1 (ex4) was constructed by cloning the two annealed oligonucleotides shootin1-f-ex4 and shootin1-r-ex4, and pT7-shootin3 (ex2) was constructed by cloning the two annealed oligonucleotides shootin3-f-ex2 and shootin3-r-ex2. The gRNAs and Cas9 mRNA were synthesized and injected into fertilized eggs as described previously^59^. The injected embryos were raised and crossed with the wild type. To screen *shootin1* and *shootin3* mutants, a T7EI assay was performed as described previously (Supplementary Fig. S6)^59,60^. We used shootin1-5′ and shootin1-3′ primers for *shootin1*, and shootin3-5′ and shootin3-3′ primers for *shootin3* (Supplementary Table S1). The PCR products were sequenced using an ABI PRISM3130 (Applied Biosystems). As shown in Supplementary Fig. S10, RT-PCR analyses confirmed that there is no detectable expression of *shootin1* in *shootin1* single mutant or *shootin1*;*shootin3* double mutant embryos. In addition, the expression of *shootin3* was undetectable in *shootin3* single mutant and *shootin1*;*shootin3* double mutant embryos.

### Genotyping

PCR-based genotyping was performed to identify *shootin1* mutants. Primers shootin1 (ex3)-wt and shootin1 (ex3)-5′ were used for screening the wild-type allele of shootin1 exon 3, and primers shootin1 (ex3)-mt and shootin1 (ex3)-5′ were used for screening the mutant allele of *shootin1* exon 3. To identify *shootin3* mutations, T7EI-mediated genotyping was performed using the primers shootin3 (ex3)-5′ and shootin3 (ex3)-3′ and two different T7EI assays. The first T7EI assay was performed as described previously (Supplementary Fig. S6a)^59^. In the second T7EI assay, PCR products obtained from samples were mixed with those from the wild type before denaturation at 94 °C for 3 min, annealing at room temperature and digestion of the annealed products with T7EI (Supplementary Fig. S6b)^60^. The first T7EI assay distinguished heterozygous fish from wild-type and homozygous fish, and the second T7EI assay distinguished between homozygous and wild-type fish (Supplementary Fig. S6c)^60^.

### Microinjection

Microinjection was performed as described previously^59^. Fertilized eggs were injected with gRNAs (50 pg/embryo) and Cas9 mRNA (300 pg/embryos). In rescue experiments, fertilized eggs were injected with *shootin1* mRNA (25 pg/embryo) and *shootin3* mRNA (25 pg/embryo).

### Tissue labeling and microscopy

Embryos were fixed in 4% paraformaldehyde (PFA) in phosphate-buffered saline (PBS: 137 mM NaCl, 2.7 mM KCl, 10 mM Na_2_HPO_4_, 1.76 mM KH_2_PO_4_, pH 7.4) overnight at 4 °C. The numbers of cells in the neuromasts and PLLP were counted by DAPI staining as described previously^18,48,49^. EdU incorporation was performed as described previously^47^. Embryos were dechorionated and 30.5-hpf embryos were immersed in 500 μM EdU solution on ice for 30 min. After washing, embryos were incubated at 28.5 °C for 1 h and fixed at 32 hpf in 4% PFA overnight at 4 °C. The EdU signals were detected using a Click-iT EdU Alexa Fluor488 Imaging Kit (Invitrogen), according to the manufacturer’s instructions. Embryos were embedded in 0.5–1% low melting point agarose (Invitrogen). Confocal images were captured with a Zeiss LSM700 or Zeiss LSM710 microscope and processed using Adobe Photoshop Elements 12 and Fiji^57^.

### Statistical analysis

Results are expressed as mean ± standard error (SEM). Statistical analyses were performed with GraphPad Prism 7. Statistical significance was determined by the two-tailed unpaired Student’s *t*-test. For multiple comparisons, we used one-way ANOVA with Tukey’s *post hoc* test.

## Supplementary information

Supplementary information

Movie 1

Movie 2

Movie 3

Movie 4

Movie 5

Movie 6

Movie 7
