# Supplementary material for: Shootins mediate collective cell migration and organogenesis of the zebrafish posterior lateral line system
Source: Sci Rep. 2019 Aug 21;9:12156. doi: 10.1038/s41598-019-48585-4 (PMC6704158; doi:10.1038/s41598-019-48585-4)
Supplement: Supplementary file 1 — Supplementary information [file 41598_2019_48585_MOESM1_ESM.pdf]

Supplementary information

Shootins mediate collective cell migration and organogenesis of the zebrafish posterior lateral line system

Akihiro Urasaki, Seiya Morishita, Kosuke Naka, Minato Uozumi, Kouki Abe, Liguo Huang, Emiko Watase, Osamu Nakagawa, Koichi Kawakami, Takaaki Matsui, Yasumasa Bessho & Naoyuki Inagaki

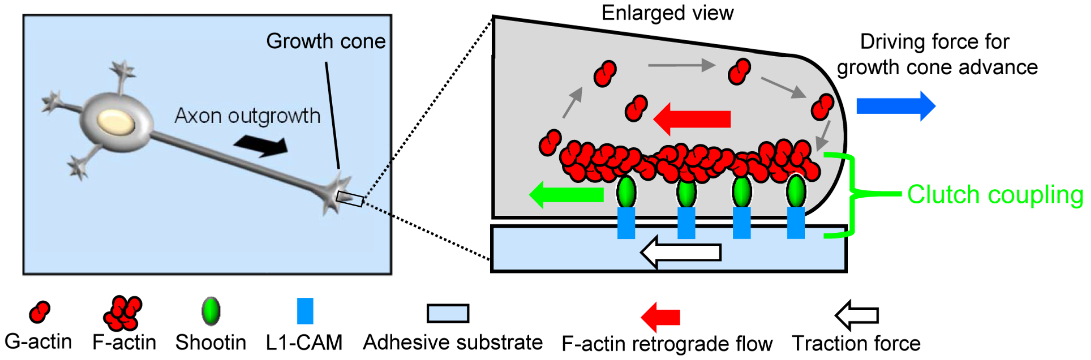

Supplementary Figure S1. Mechanism of migration of the axonal growth cone.
F-actins polymerize at the leading edge of the axonal growth cone and depolymerize proximally, thereby inducing retrograde flow of F-actins (red arrow). Shootin1 couples mechanically the F-actin retrograde flow and extracellular adhesive substrates, a process called "clutch coupling", thereby transmitting the force of F-actin flow (red arrow) to the substrates as a traction force (white arrow)[1-3]. The driving force for growth cone advance (blue arrow) is produced as a counterforce to the traction forces exerted on the substrate (white arrow).

```
                                                        CC1
Mouse shootin1a     MNSSDEEKQLQLITSLKEQAIGEYEDLRAENQKTKEKCDKIRQERDEAVKKLEEFQKISHMVIEEVNFMQNHLEIEKTCRESAEALATKL  90
Zebrafish shootin1  MASKGKKNKVRTNSDLSNQVLLQYESLQKEHEKIKKECKKLQEERDEALRKLNEFESVSHRVIEEVNSIQENLEIEKTCRESVEALASKL  90
Zebrafish shootin2  ------------MWSLESVSDSEEDNTPSSEDERDFECQILEKERDEANEKLSRMEEASSHLLKELDVLEMQFQIERSCRETAEAYALKV  78
Zebrafish shootin3  ------MDTTPLSAGCVSEMEAGGDAEEGEARFGSTECQRLTAERDEAERQLKHIKRVSQMVIEEVNVLQTQLEIEKSCRENAEALATKL  84
                                                         *     *****   *      *     *         **  ***  ** * *

                                                                                      CC2
Mouse shootin1a     NKENKTLKRIS---MLYMAKLGPDVITEEINID-DDDPATDTDAAAETCVSVQCQKQIKELRDQIVSVQEEKKVLAIELENLKSKLGEVM 176
Zebrafish shootin1  NRQNRSLKRKS---MLYMSRLAADVVAE-ISID-DEDDEDAHEEEAGVCSSSHCHIVITELRDKLEEILATKKQLMIDLETTREQLSKTR 175
Zebrafish shootin2  SKDFKVLKRQSQALLPLIQDMPENLSALNLDSEADLSSDSVPAEDSSEDPLLMSQNQIRELQSSVDRLLGEKIQLCAQVDLLKKEKEDLK 168
Zebrafish shootin3  NCENRKLKYLS---LSSRPCLD-ELLPSISDCI-SLEEEVEQQDHSSDSFS-QYKQQVKDLQETVSSLLEEKKQLACQLQEQQRQIKELT 168
                          **   *                                                 *          *  *

Mouse shootin1a     EEVNKVKQEKAVLNSEVLEQRKVLEKCNRVSMLAVEEYEELQVNLELEKDLRKKAESFAQEMFIEQNKLKRQSHLLLQSSLPDQQLLKAL 266
Zebrafish shootin1  QELLKEKHDNTVLIAETFQQKKLLGKYNRVSQYALDEFESLQEDLKLERDLRSEAEKFAHEMLIEQKKLKRQSQVLVQSISVGEALQKAL 265
Zebrafish shootin2  EKLVLEIGEKEALLKKLSKQSRTVNKIKRVSQLVTDEFTEISQKLEMEQDLRQHAEVFAHQMLVKQRETQRQ----IQNAETEKQLQQTL 258
Zebrafish shootin3  ALREKEQAEMKELYKTIDQQSKTIKRFNRVSMVATTEYENMKEQLDLEQSLRQKAETYAHEMMVKQKEANRQSMILLQQADPSIQLLKAL 258
                                *      *        ***     *       *  *  **  **  *  *   *    **     *       *   *
        CC3
Mouse shootin1a     DENAKLIQQLEEERIQHQKKVKELEERLENEALHKEIHNLRQQLELLEDDKRELEQKYQSSEEKARNLKHSVDELQ-----------KRV 345
Zebrafish shootin1  AEISTLTHTLEKQRLEHQQQVKALEEQVNSSEVKKQLTALQRQTDLLEEERKEWQHKHTKAETEAKDLRFTVEELK-----------KKL 344
Zebrafish shootin2  SQVSDISRALEEIRLCYQTQMS--QTAAEDLNSLSDLTAIRTKLEISERERSETETQLRDSQQAVSALQEQIKLLQDKLREKEQIPLIII 342
Zebrafish shootin3  EDVANVTKTLEEERMQHQEKVKALETELEQCALRKQLVQLQRQLEILDEEKKETEGRLQEEEKKNTILEEKVKDLQEA-RTSSDSASGPS 347
                             **   *  *                                  *              *      *
                      Proline-rich domain
Mouse shootin1a     NQSENSV-----------PPPPPPPPPLPPPPP-NPIRSLMSMIRKRSHPSGNSAKKEKTTQPETAEEVTDLKRQAVEEMMDRIKKGVHL 423
Zebrafish shootin1  QQVSNPPTAAPAPP--PPPPPPPPPPPPSSSSS-NPLSSLLSILRKKKDVSTEIALVEKDS-SEKSPE-KDVRQQAVDEMMLRIKKGVQL 431
Zebrafish shootin2  DQSEETNDNPSDPPLLPPPPSPPPPPPPPPPPPPPPSSFVTDPLKALRNRKKAGENTTQTAKPTITE---DMKARAVDEMMERIKKGIVL 432
Zebrafish shootin3  PGTAPPPAPPPQPP--PPPPPPPPPPPPPPPSRCNPLSSLIAIMRKSSKGGKGTPKLEQAPVNEAVD---DVKVKAVNEMMERIKHGVVL 435
                                      ** ******        *                                   *    ** *** *** *  *

Mouse shootin1a     RPV-NQTA-R-----------PKAKPDSLKGSESAVDELKGILASQ-------------------------------------------- 456
Zebrafish shootin1  RPV-SQTTNR-----------VRPGPKEPTASNSAIQELQGILNTVKRPGPSSSPGPRPPSPSEKSELEKALQRRREAVKSAKNNTNSSS 507
Zebrafish shootin2  KPVLRPPHVASEDENAWKEQRSENRKSAVLELQEMLDNVRRSAPRRVESSKRFSRNVGEAELQMVLQRRRRAMGDDKVTPPSPTKPKDSP 519
Zebrafish shootin3  RPVKSQDTKRFGTKLPSPVTAAAAAAVEEKHQESAMEELKGILETVKKS-PSRGFQEVAHVKKD-SELEVILRRRRKQA-CDTGAEDNGQ 519
                     **

Mouse shootin1a     ------------------------------------------------------------------------------------------ 456
Zebrafish shootin1  VVDLTQIKQTRSEPGQNTGDQETLRHTTTTICTEQLS----------------------------------------------------- 544
Zebrafish shootin2  VSSSSPWAGESGSAPVLRRLQQNREKRVSRIKASESIIWEEK------------------------------------------------ 561
Zebrafish shootin3  LSKVSSSNSLNGRHSSDSGKDTEGPSSSSLSGSERGSRTSSDSGREPAVVQQSSDSGMESKGAAQTDSDCRSLTEKEPIEPITNGCCSGE 579

Mouse shootin1a     ----------------------------------------------                                             456
Zebrafish shootin1  ----------------------------------------------                                             544
Zebrafish shootin2  ----------------------------------------------                                             561
Zebrafish shootin3  GQDGDPEKWTGPNGIGHSVAHGDLQTTISSSAQSPNAANSSTDAEC                                             655
```

Supplementary Figure S2. Multiple sequence alignment of shootin proteins.

Amino acid sequences of zebrafish shootin1, shootin2 and shootin3 are aligned with mouse shootin1. Identical residues are shown by asterisks. Red underlines indicate coiled-coil domains predicted by SMART[4]. Blue underlines indicate proline-rich domains. Arrowheads indicate putative sites for phosphorylation by Pak1[5].

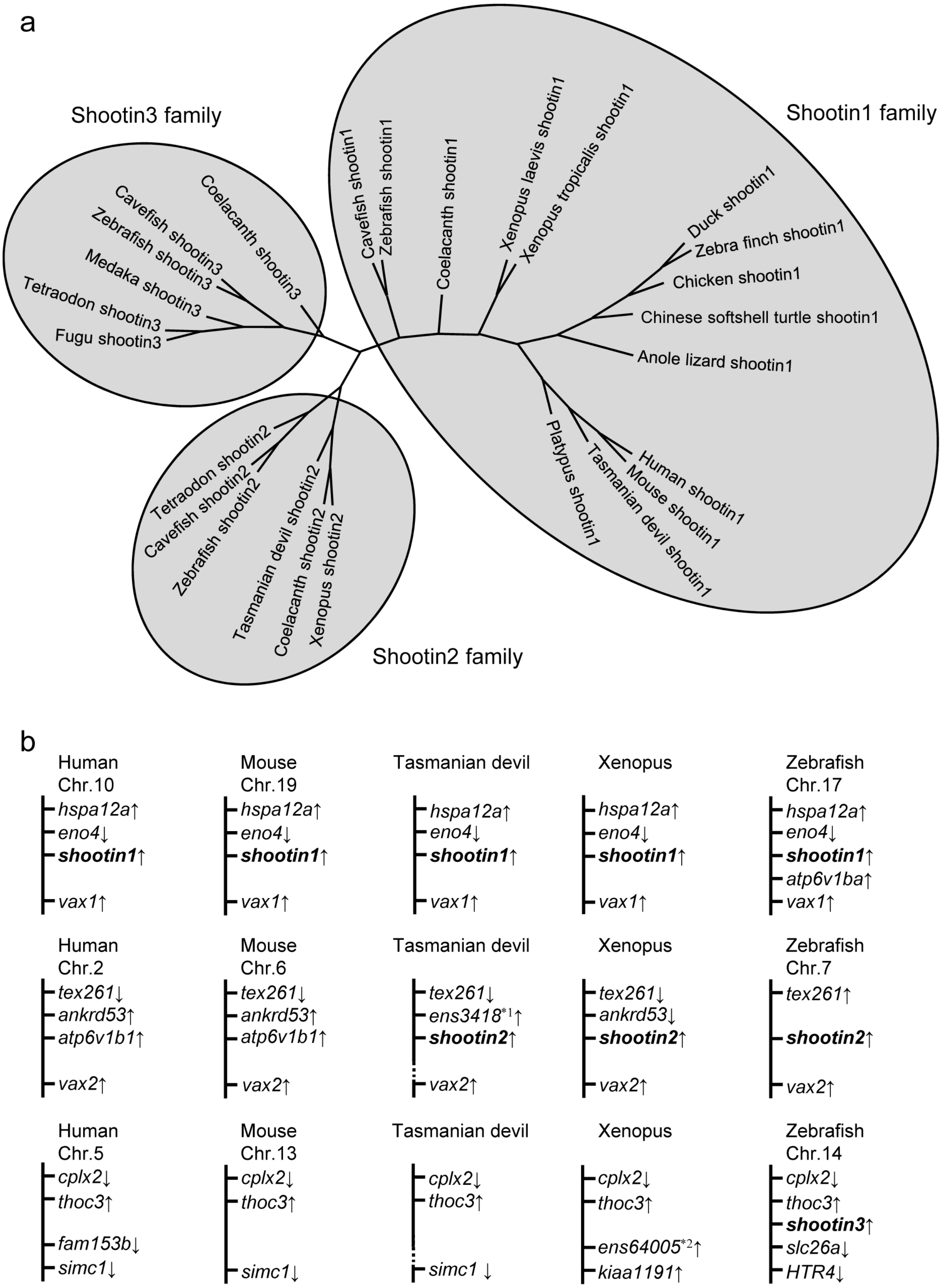

Supplementary Figure S3. Phylogenetic tree and synteny analysis of vertebrate shootin genes.

(a) Phylogenetic tree of vertebrate shootins. Accession numbers of protein sequences used in the phylogenetic analysis are listed in Supplementary Table S2. (b) Synteny analysis of vertebrate shootin genes in human, mouse, Tasmanian devil, *Xenopus* and zebrafish genome assemblies. Arrows indicate the translational orientation of genes. Dashed lines represent synteny breaks. Loci containing shootin genes in the Tasmanian devil and *Xenopus* genomes are mapped onto sequence scaffolds. ens3418[*1]: ENSSHAG0000003418; and ens64005[*2]: ENSXETT00000064005.

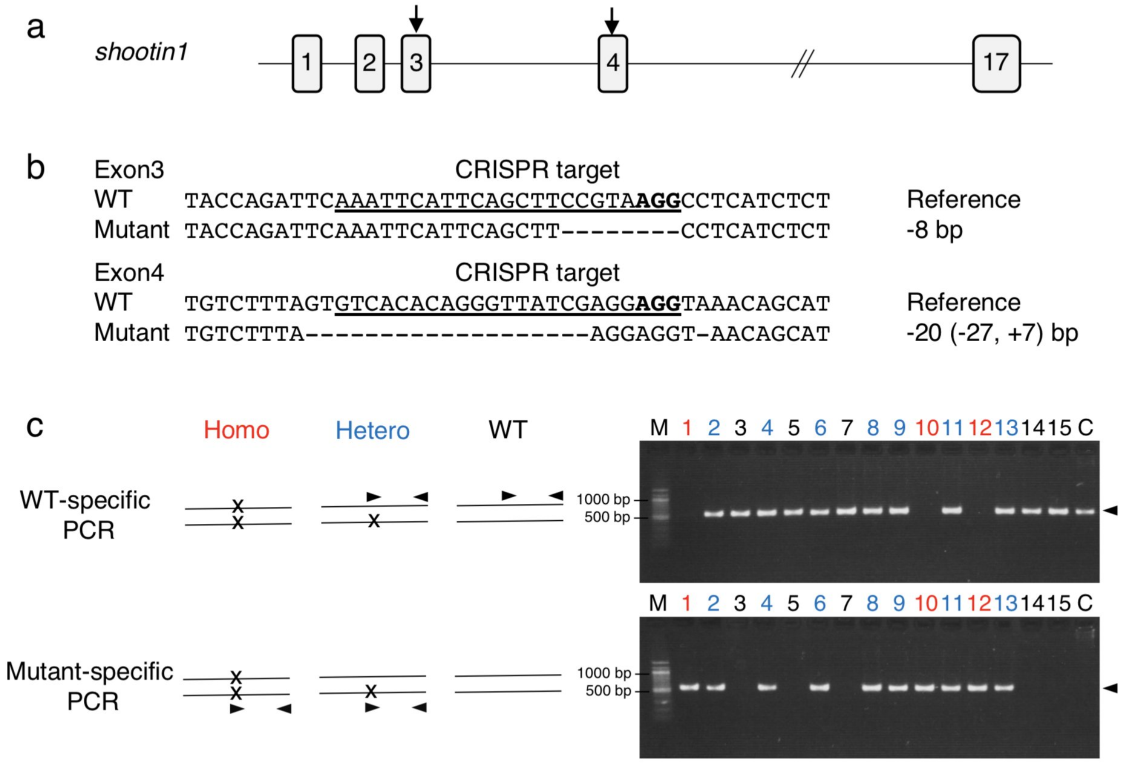

Supplementary Figure S4. Generation of a *shootin1* mutant using the CRISPR/Cas9 system.

(a) Schematic representation of the genomic structure of the *shootin1* gene. Numbers in boxes indicate the exon numbers of *shootin1*. Arrows indicate the positions of the CRISPR targets in *shootin1*. (b) DNA sequences of the *shootin1* CRISPR target in the wild type and *shootin1* mutant. Underlines indicate the sequences of the CRISPR target sites. Bold letters indicate protospacer adjacent motif (PAM) sequences. The *shootin1* mutant zebrafish carried two mutations in exon3 and exon4 because two *shootin1* gRNAs were injected with Cas9 mRNA into fertilized eggs. (c) PCR-based genotyping of *shootin1*. PCR reactions were performed using wild-type-specific primers and mutant-specific primers. The different colors of lane numbers indicate different genetic backgrounds: homozygous (red), heterozygous (blue) and wild type (black). Lane M, DNA marker; lane C, PCR products obtained using wild-type genomic DNA template as controls.

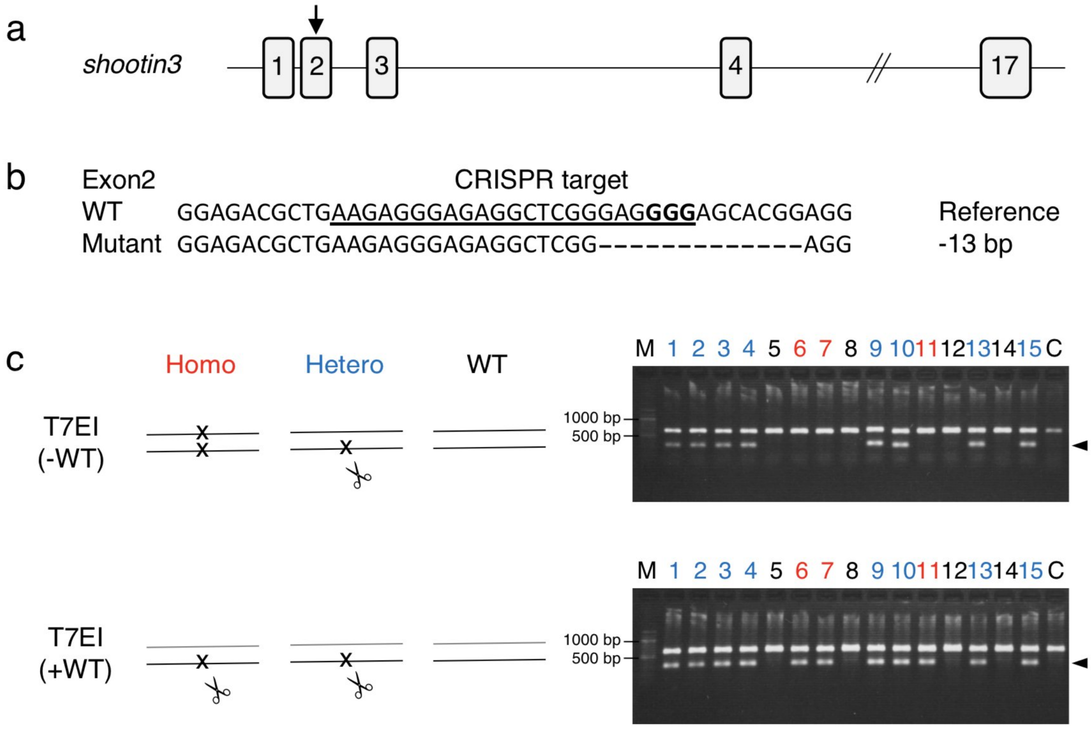

Supplementary Figure S5. Generation of a *shootin3* mutant using the CRISPR/Cas9 system.

(a) Schematic representation of the genomic structure of the *shootin3* gene. Numbers in boxes indicate the exon numbers of *shootin3*. The arrow indicates the position of the CRISPR target in *shootin3*. (b) DNA sequences of *shootin3* in the wild type and *shootin3* mutant. The underline indicates the sequence of the CRISPR target site. Bold letters indicate the PAM sequence. (c) T7EI-based *shootin3* genotyping. PCR reactions were performed using shootin3-specific primers. PCR products were denatured and reannealed without wild-type PCR products (–WT) or with wild-type PCR products (+WT). T7EI recognizes and cleaves mismatched bases in double-stranded DNA. The annealed double-strand DNAs were treated with T7EI and analyzed by electrophoresis in a 3% agarose gel. Arrowheads indicate T7EI-digested bands. The different colors of lane numbers indicate different genetic backgrounds: homozygous (red), heterozygous (blue) and wild type (black). A detailed explanation of T7EI-based genotyping is described in Supplementary Figure S6 and its legend. Lane M, DNA marker; lane C, PCR products obtained using wild-type genomic DNA template as controls.

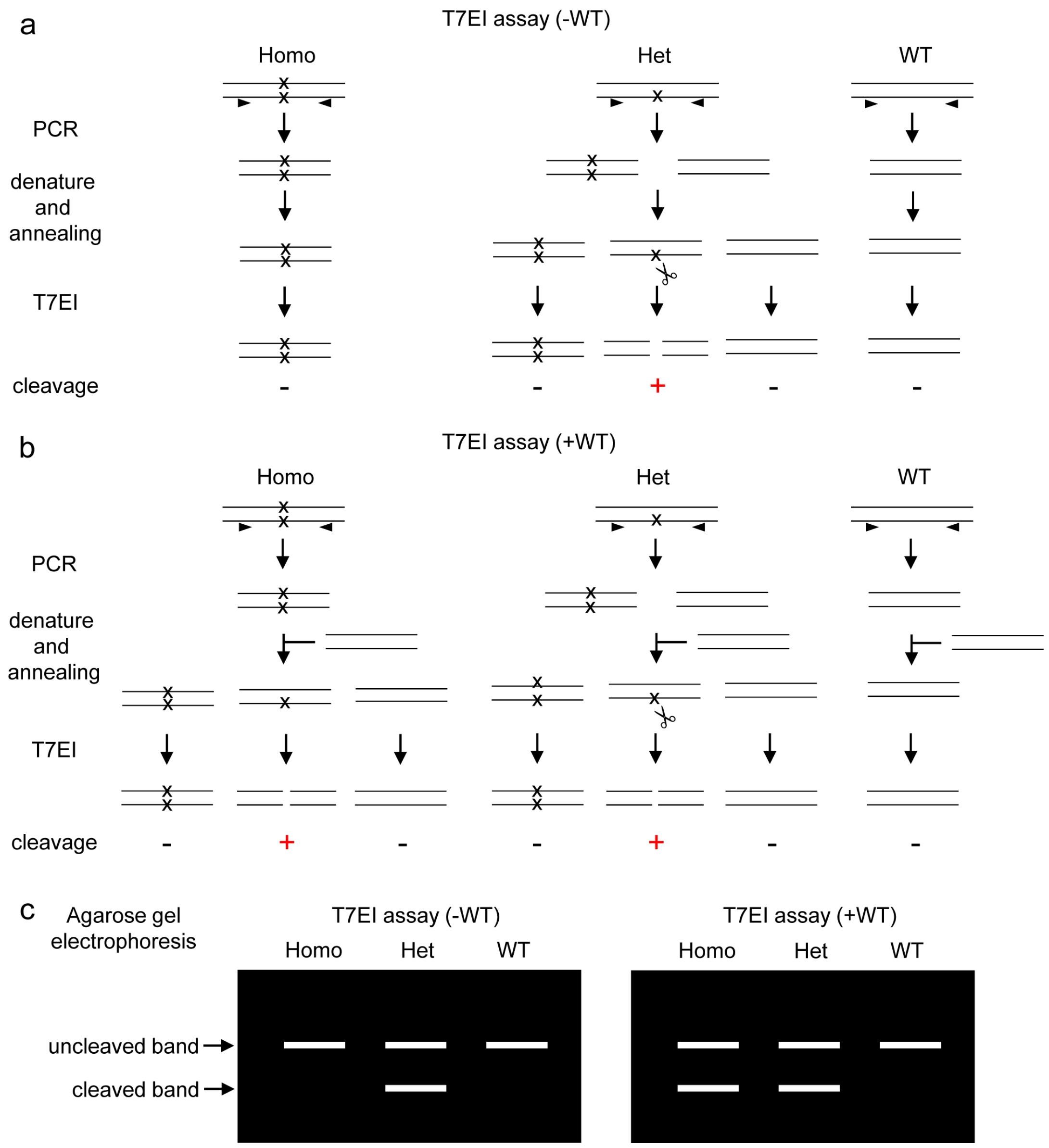

Supplementary Figure S6. Schematic overview of T7EI-based genotyping.

(a) T7EI assay without wild-type PCR products (-WT). PCR reactions are performed using *shootin3*-specific primers, and the PCR products are then denatured and reannealed without wild-type PCR products (–WT). The annealed double-stranded DNAs are treated with T7EI, which recognizes and cleaves mismatched bases in double-stranded DNA (scissors). The T7EI assay (-WT) can therefore distinguish heterozygous fish from homozygous and wild-type fish. (b) T7EI assay with wild-type PCR products (+WT). PCR reactions are performed using *shootin3*-specific primers. The PCR products are mixed with wild-type PCR products, and then denatured and

reannealed. The annealed double-stranded DNAs are treated with T7EI. The T7EI assay (+WT) can thus distinguish between homozygous/heterozygous fish and wild-type fish. (c) Expected results of T7EI-based genotyping. The T7EI-treated DNAs of (a) and (b) are analyzed by electrophoresis on a 3% agarose gel. Cleaved bands can be detected only in heterozygous fish (Het) in the T7EI assay (-WT), whereas the cleaved bands can be detected in both homozygous fish (Homo) and heterozygous fish (Het) in the T7EI assay (+WT). Arrowheads in (a) and (b) indicate PCR primers.

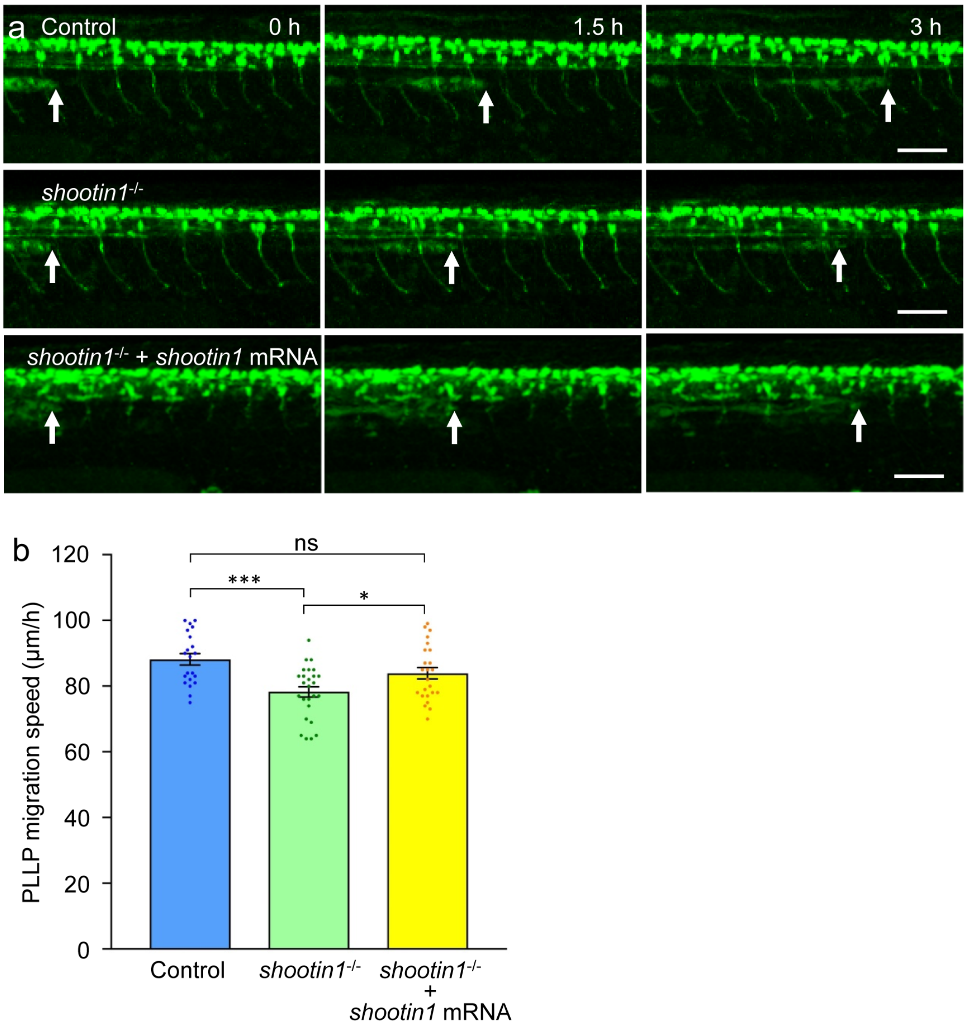

Supplementary Figure S7. *shootin1* mutants display reduced migration speed of the PLLP.

(a) Representative time-lapse images of wild-type control, *shootin1*$^{-/-}$ single mutant, and *shootin1* mRNA-injected *shootin1*$^{-/-}$ single mutant embryos carrying the SAIGFF213A;UAS:GFP construct. Arrows indicate the leading edges of PLLPs. Scale bars: 50 µm. (b) Migration speeds of PLLP in wild-type control (n = 21), *shootin1*$^{-/-}$ single mutant (n = 26) and *shootin1* mRNA-injected *shootin1*$^{-/-}$ single mutant (n = 24) embryos at 32-38 hpf obtained from the analyses in (a). *shootin1* mRNA was injected into the *shootin1*$^{-/-}$ single mutant embryos. Data for the uninjected wild-type and *shootin1*$^{-/-}$ single mutant embryos in (b) are shared with those in Figure 3c. Data for (b) represent mean ± SEM. Statistical significance of the differences is indicated with asterisks (***, $P < 0.01$; *, $P < 0.05$; ns, nonsignificant).

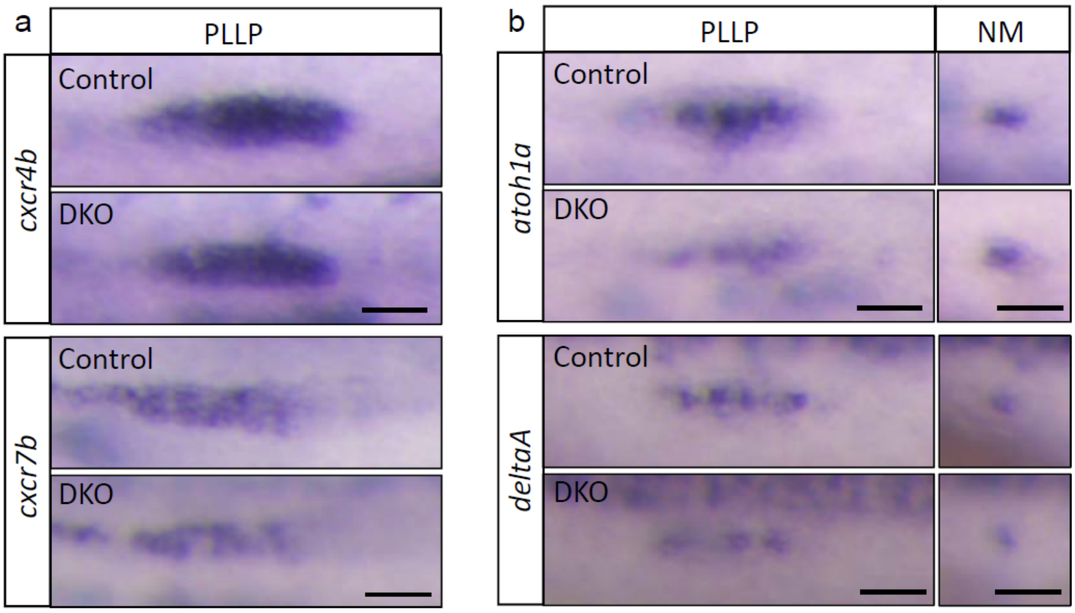

Supplementary Figure S8. *In situ* hybridization of wild-type and *shootin1*;*shootin3* double mutant embryos at 32 hpf.

(a) *In situ* hybridization of *cxcr4b* and *cxcr7b* in wild-type control (control) and *shootin1*;*shootin3* double mutant (DKO) PLLP at 32 hpf. (b) *In situ* hybridization of *atoh1a* and *deltaA* in wild-type control and *shootin1*;*shootin3* double mutant (DKO) PLLP and neuromasts (NM) at 32 hpf. Scale bars: 25 µm.

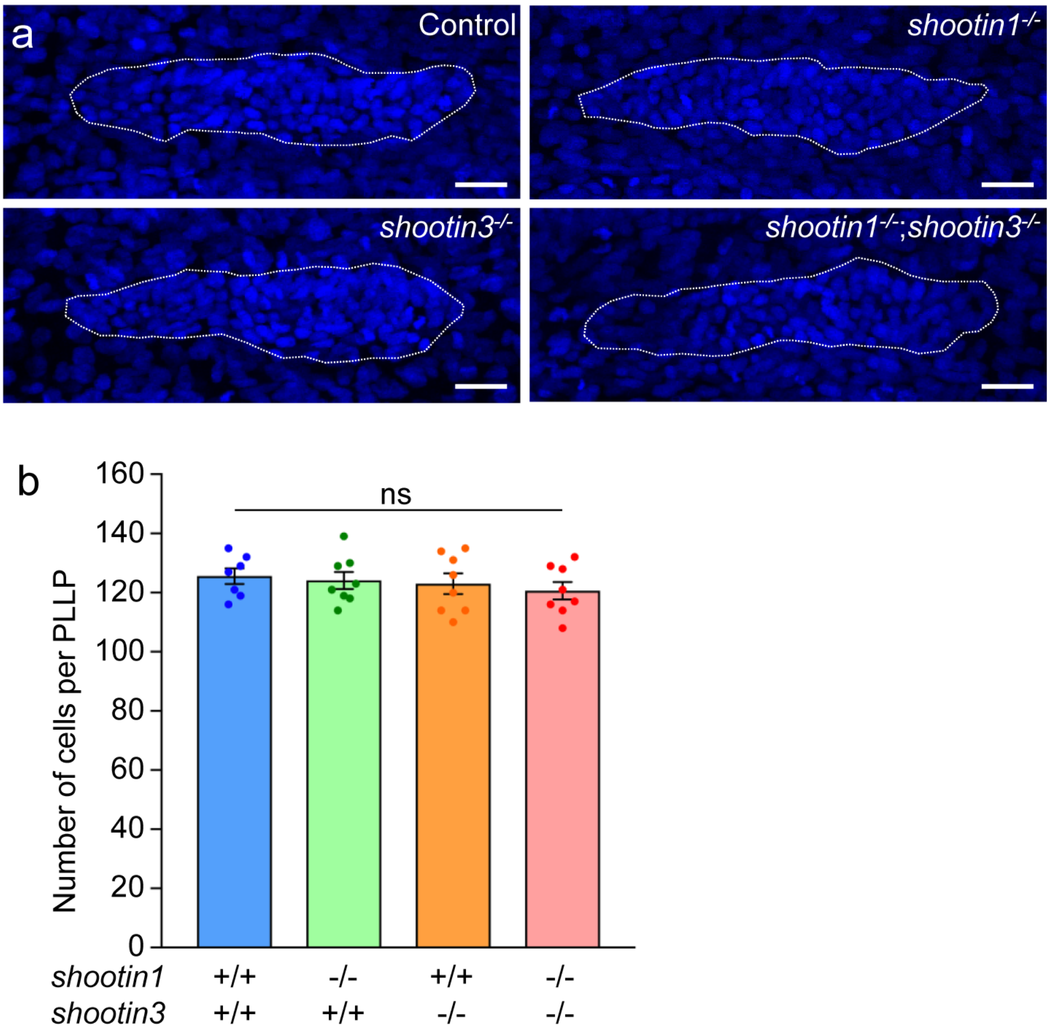

Supplementary Figure S9. Mutations in *shootin1* and *shootin3* do not affect cell number in the PLLP at 24 hpf.

(a) Representative images of DAPI-stained PLLP in wild-type control, *shootin1*$^{-/-}$ single mutant, *shootin3*$^{-/-}$ single mutant and *shootin1*$^{-/-}$;*shootin3*$^{-/-}$ double mutant embryos at 24 hpf. Dotted lines indicate the areas of PLLP, in which small nuclei of PLLP cells cluster. Scale bars: 20 μm. (b) The number of cells in the PLLP of wild-type control (n = 7), *shootin1*$^{-/-}$ single mutant (n = 8), *shootin3*$^{-/-}$ single mutant (n = 8) and *shootin1*$^{-/-}$;*shootin3*$^{-/-}$ double mutant (n = 8) embryos at 24 hpf was analyzed, using DAPI staining.

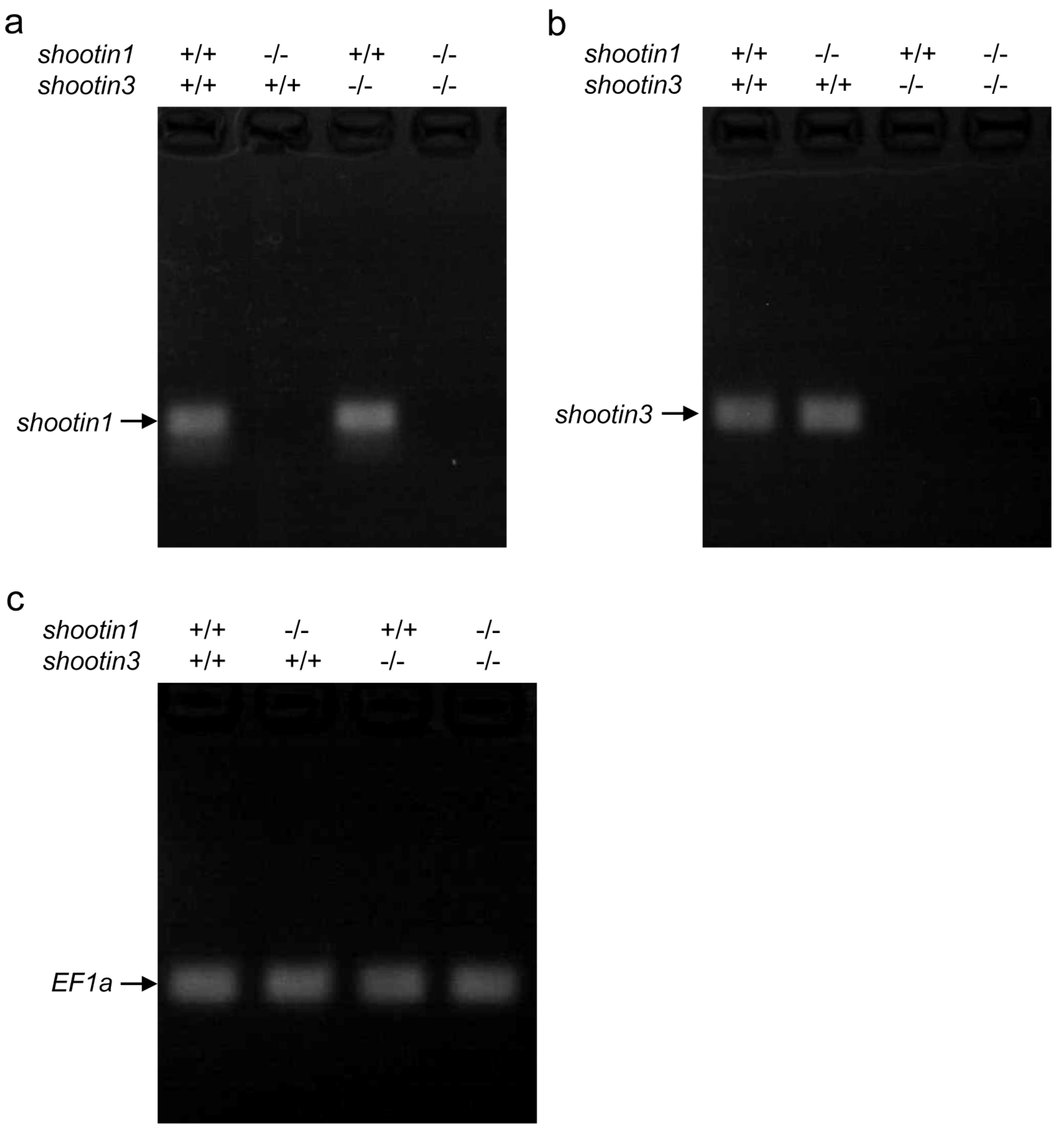

Supplementary Figure S10 RT-PCR analysis of *shootin1*, *shootin3* and *EF1a* transcripts.

(a) RT-PCR using *shootin1*-specific primers. (b) RT-PCR using shootin3-specific primers. (c) RT-PCR using *EF1a*-specific primers (positive control). Total RNA was prepared from wild-type, *shootin1* single mutant, *shootin3* single mutant and *shootin1*;*shootin3* double mutant embryos at 48 hpf.

Supplementary Figure S11. Full-length gel images in Figure 1b.

The full-length gel image in Figure 1b *shootin1*

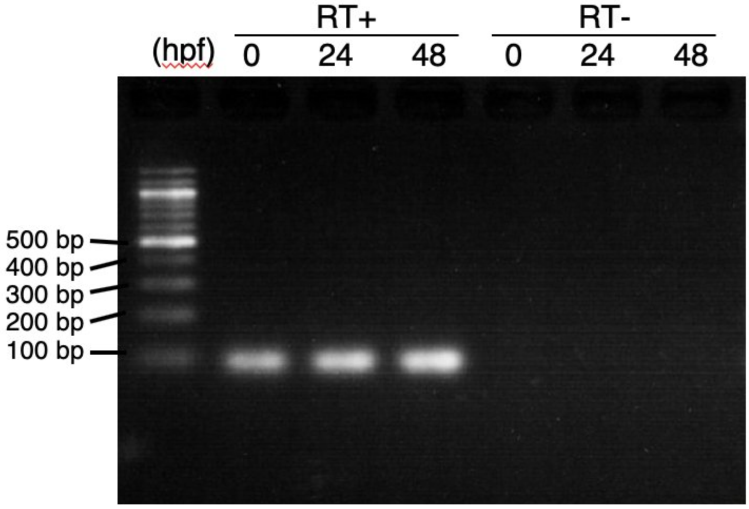

The full-length gel image in Figure 1b *shootin2*

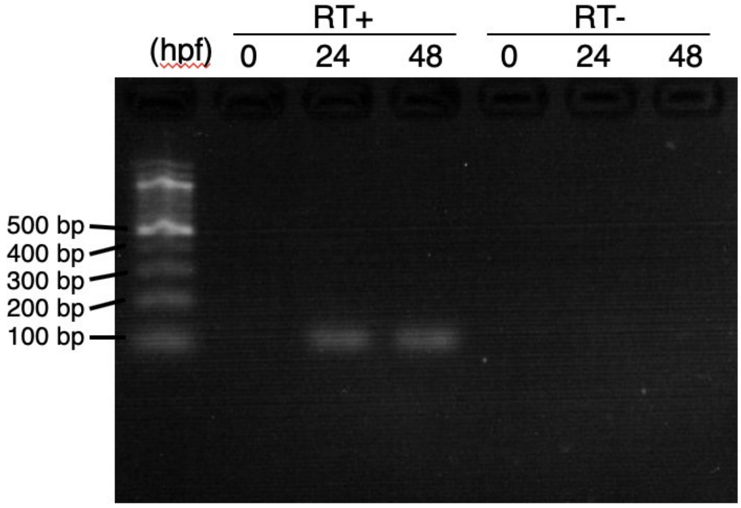

The full-length gel image in Figure 1b *shootin3*

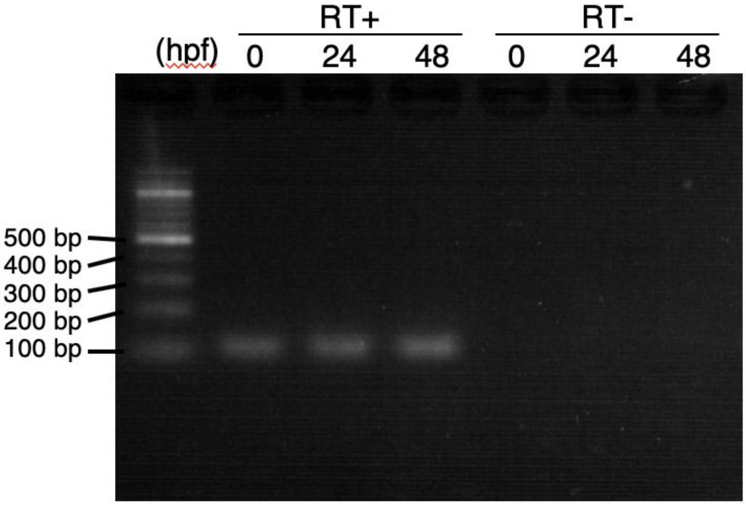

The full-length gel image in Figure 1b *EF1a*

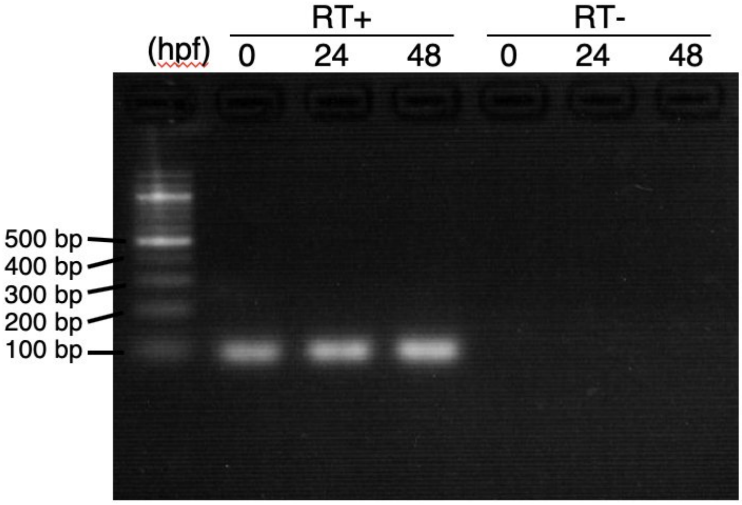

Supplementary Table S1. Oligonucleotide list.

Name	Sequence (5' to 3')
AP	GGCCACGCGTCGACTAGTACTTTTTTTTTTTTTTTTT
shootin1-h-Ba-Ko	AAAGGATCCGCCACCATGGCGTCAAAAGGAAAGAAGAA
shootin1-t-Xb-No	TTTGCGGCCGCTCTAGACTATGAGAGCTGCTCAGTGCATAT
shootin2-h-Ba-Ko	TTTGGATCCGCCACCATGTGGAGTCTTGAATCAGTATCAG
shootin2-t-Xb-No	TTTGCGGCCGCTCTAGATCACTTTTCTTCCCATATAATGGAC
shootin3-h-Ba-Ko	AAAGGATCCGCCACCATGGATACGACGCCGCTGTCTGC
shootin3-t-Sp	TTTACTAGTTTAACACTCGGCGTCTGTGCT
pCS2-5'-out-Ba	TTTGGATCCTGCAAAAAGAACAAGTAGCTTGTA
pCS2-3'-out-Ba-Sp-Bg-Xb	TTTGGATCCGGGACTAGTGGGAGATCTGGGTCTAGATAAG
	CTAGAACTATAGTGAGTCGTATTACGTAG
EGFP-h-Bg-Ko	TTTAGATCTGCCACCATGGTGAGCAAGGGCGAGGAGCTG
EGFP-t-Sp	TTTACTAGTCTTGTACAGCTCGTCCATGCCG
shootin1-h-Ba	AAAGGATCCATGGCGTCAAAAGGAAAGAAG
shootin2-h-Ba	TTTGGATCCATGTGGAGTCTTGAATCAGTATCAG
shootin3-h-Ba	AAAGGATCCATGGATACGACGCCGCTGTCT
shootin1-t-Sa	TTTGTCGACTGAGAGCTGCTCAGTGCATAT
shootin2-t-Sa	TTTGTCGACCTTTTCTTCCCATATAATGGAC
shootin3-t-Sa	TTTGTCGACACTAGTTTAACACTCGGCGTCTGTGCT
shootin1-rt-f	ATTGCAAGAGGAGAGAGATGAGG
shootin1-rt-r	CTCCTGAATGCTGTTTACCTCCT
shootin2-rt-f	TCCTCAGAAGATGAAAGAGACTTTG
shootin2-rt-r	CCTTCAGCAGATGAGAAGAGG
shootin3-rt-f	TGTGTGAGTGAGATGGAGGC
shootin3-rt-r	AGCCTCATCTCTCTCTGCAGT
EF1a-rt-f	AGCGGTACTACTCTTCTTGATGC
EF1a-rt-r	TTGTACACATCCTGAAGTGGCA
shootin1-r2	TCTGCCAGAGCTTTCTGTAGAGCTT
shootin2-h4	TTTGGATCCATGTGGAGTCTTGAATCAGTATCAG
shootin2-r4	TGGGGAAGGAGGAGGCGGCAGTA
shootin3-f	GGAAGTCTTCCAAGGGAGGTAAAGG
shootin1-f-ex3	TAGGAAATTCATTCAGCTTCCGTA
shootin1-r-ex3	AAACTACGGAAGCTGAATGAATTT
shootin1-f-ex4	TAGGGTCACACAGGGTTATCGAGG
shootin1-r-ex4	AAACCCTCGATAACCCTGTGTGAC

shootin3-f-ex2	TAGGAAGAGGGAGAGGCTCGGGAG
shootin3-r-ex2	AAACCTCCCGAGCCTCTCCCTCTT
shootin1 (ex3)-5’	TAATTACCCTAACCTTAACCTAGTC
shootin1 (ex3)-3’	TCTAGGGGAAATGTAGATAGAGCGA
shootin1 (ex3)-wt	TAGGAAATTCATTCAGCTTCCGTA
shootin1 (ex3)-mt	ATTCAAATTCATTCAGCTTCCTCA
shootin1 (ex4)-5’	ACCTTGTGTCCACCATATAAACAGT
shootin1 (ex4)-3’	ATTAGCTGAACCAGAACACCACAGA
shootin3 (ex2)-5’	ATGAGCCAAGCCTTCGCCATAGTAG
shootin3 (ex2)-3’	GGCCACATACAATGTGCCTCAAAGC
atoh1a-F	ACGAGCTGCGCAGTGTCATC
atoh1a-R	CATGGCGTAGGGGTCTGGTC
cxcr4b-F	CGCGTGGGGAGACTTATTGC
cxcr4b-R	GCGGGTAAGTAAGCTCGCAGA
cxcr7b-F	TCTTCAGTGTCAACCTCTTCAGCA
cxcr7b-R	GCCTTCATGAGGTCGTATCGGTAG
deltaA-F	GAAGATCGACCACTGCTCTTCCA
deltaA-R	ATTCTTAACCGGCGCCACTC

Supplementary Table S2. Accession numbers of protein sequences used in the phylogenetic analysis.

Name	Accession numbers
Danio rerio (zebrafish) shootin1	LC310870
Danio rerio (zebrafish) shootin2	LC310871
Danio rerio (zebrafish) shootin3	LC310872
Homo sapiens (human) shootin1a	SHTN1-001 ENST00000615301.4
Mus musculus (mouse) shootin1a	SHTN1-202 ENSMUST00000163821.1
*Sarcophilus harrisii (*Tasmanian devil) shootin1	ENSSHAT00000020502.1
Sarcophilus harrisii (Tasmanian devil) shootin2	ENSSHAT00000004495.1
Gallus gallus (chicken) shootin1	ENSGALT00000068208.1
Anas platyrhynchos (duck) shootin1	SHTN1-201 ENSAPLT00000010851.1
Taeniopygia guttata (zebra finch) shootin1	SHTN1-201 ENSTGUT00000011512.1
Ornithorhynchus anatinus (platypus) shootin1	ENSOANT00000022297.3
Anolis carolinensis (anole lizard) shootin1	SHTN1-201 ENSACAT00000010191.3
Pelodiscus sinensis (Chinese softshell turtle)	SHTN1-201 ENSPSIT00000015476.1
Xenopus laevis (frog) shootin1	XM_018224676.1 (LOC108695700)
Xenopus tropicalis (frog) shootin1	XP_004915784.1
Xenopus laevis (frog) shootin2	LOC100486585.L
Coelacanthiformes (coelacanth) shootin1	SHTN1-201 ENSLACT00000011686.1
Coelacanthiformes (coelacanth) shootin2	ENSLACT00000006377.1
Coelacanthiformes (coelacanth) shootin3	ENSLACT00000008805.1
Tetraodon nigroviridis (tetraodon) shootin2	si:ch211-1o7.3-201 ENSTNIT00000006279.1
Tetraodon nigroviridis (tetraodon) shootin3	si:dkey-280e21.3-201 ENSTNIT00000016149.1
Takifugu rubripes (fugu) shootin3	si:dkey-280e21.3-201 ENSTRUT00000007489.1
Oryzias latipes (medaka) shootin3	ENSORLT00000014420.1
Astyanax mexicanus (cave fish) shootin1	SHTN1-201 ENSAMXT00000011964.1
Astyanax mexicanus (cave fish) shootin2	si:ch211-1o7.3-201 ENSAMXT00000005924.1
Astyanax mexicanus (cave fish) shootin3	si:dkey-280e21.3-201 ENSAMXT00000011022.1

Shootin1 in *Oryzias latipes*, *Tetraodon nigroviridis* and *Takifugu rubripes* and shootin2 in *Oryzias latipes*, *Takifugu rubripes* and *Xenopus tropicalis* were not annotated.

Movie 1. Fluorescent speckles of AcGFP-shootin1 and mRFP-actin in XTC fibroblasts. Images were acquired at 5-s intervals.

Movie 2. Fluorescent speckles of AcGFP-shootin2 and mRFP-actin in XTC fibroblasts. Images were acquired at 5-s intervals.

Movie 3. Fluorescent speckles of AcGFP-shootin3 and mRFP-actin in XTC fibroblasts. Images were acquired at 5-s intervals.

Movie 4. Time-lapse imaging of a wild-type control embryo of zebrafish carrying the SAIGFF213A;UAS:GFP construct. Movie starts and ends at approximately 32 and 38 hpf, respectively. Images were acquired at 20-min intervals.

Movie 5. Time-lapse imaging of a *shootin1*$^{-/-}$ single mutant embryo of zebrafish carrying the SAIGFF213A;UAS:GFP construct. Movie starts and ends at approximately 32 and 38 hpf, respectively. Images were acquired at 20-min intervals.

Movie 6. Time-lapse imaging of a *shootin3*$^{-/-}$ single mutant embryo of zebrafish carrying the SAIGFF213A;UAS:GFP construct. Movie starts and ends at approximately 32 and 38 hpf, respectively. Images were acquired at 20-min intervals.

Movie 7. Time-lapse imaging of a *shootin1*$^{-/-}$;*shootin3*$^{-/-}$ double mutant embryo of zebrafish carrying the SAIGFF213A;UAS:GFP construct. Movie starts and ends at approximately 32 and 38 hpf, respectively. Images were acquired at 20-min intervals.

References

1 Shimada, T. *et al.* Shootin1 interacts with actin retrograde flow and L1-CAM to promote axon outgrowth. *J Cell Biol* **181**, 817-829 (2008).

2 Kubo, Y. *et al.* Shootin1-cortactin interaction mediates signal-force transduction for axon outgrowth. *J Cell Biol* **210**, 663-676 (2015).

3 Baba, K. *et al.* Gradient-reading and mechano-effector machinery for netrin-1–induced axon guidance. *eLife* **7**, e34593 (2018).

4 Letunic, I. & Bork, P. 20 years of the SMART protein domain annotation resource. *Nucleic Acids Res* **46**, D493-D496 (2018).

5 Toriyama, M., Kozawa, S., Sakumura, Y. & Inagaki, N. Conversion of a signal into forces for axon outgrowth through Pak1-mediated shootin1 phosphorylation. *Curr Biol* **23**, 529-534 (2013).
